# The value of functional magnetic resonance imaging in the evaluation of diabetic kidney disease: a systematic review and meta-analysis

**DOI:** 10.3389/fendo.2023.1226830

**Published:** 2023-07-07

**Authors:** Ziqi Zhang, Yu Chen, Xiqiao Zhou, Su Liu, Jiangyi Yu

**Affiliations:** ^1^ Department of Endocrinology, Jiangsu Provincial Hospital of Chinese Medicine, Affiliated Hospital of Nanjing University of Chinese Medicine, Nanjing, Jiangsu, China; ^2^ The First Clinical Medical College of Nanjing University of Chinese Medicine, Nanjing, China

**Keywords:** functional magnetic resonance imaging, diabetic kidney disease, meta-analysis, fMRI, DKD

## Abstract

**Background:**

The diversity of clinical trajectories in diabetic kidney disease (DKD) has made blood and biochemical urine markers less precise, while renal puncture, the gold standard, is almost impossible in the assessment of diabetic kidney disease, and the value of functional magnetic resonance imaging in the evaluation of diabetic pathological alterations is increasingly recognized.

**Methods:**

The literature on functional magnetic resonance imaging (fMRI) for the assessment of renal alterations in diabetic kidney disease was searched in PubMed, Web of Science, Cochrane Library, and Embase databases. The search time limit is from database creation to March 10, 2023. RevMan was used to perform a meta-analysis of the main parameters of fMRIs extracted from DKD patients and healthy volunteers (HV).

**Results:**

24 publications (1550 subjects) were included in this study, using five functional MRIs with seven different parameters. The renal blood flow (RBF) values on Arterial spin labeling magnetic resonance imaging (ASL-MRI) was significantly lower in the DKD group than in the HV group. The [WMD=-99.03, 95% CI (-135.8,-62.27), *P*<0.00001]; Diffusion tensor imaging magnetic resonance imaging (DTI-MRI) showed that the fractional anisotropy (FA) values in the DKD group were significantly lower than that in HV group [WMD=-0.02, 95%CI (-0.03,-0.01), *P*<0.0001]. And there were no statistically significant differences in the relevant parameters in Blood oxygen level-dependent magnetic resonance imaging (BOLD-MRI) or Intro-voxel incoherent movement magnetic resonance imaging (IVIM-DWI).

**Discussion:**

ASL and DWI can identify the differences between DKD and HV. DTI has a significant advantage in assessing renal cortical changes; IVIM has some value in determining early diabetic kidney disease from the cortex or medulla. We recommend combining multiple fMRI parameters to assess structural or functional changes in the kidney to make the assessment more comprehensive. We did not observe a significant risk of bias in the present study.

**Systematic review registration:**

https://www.crd.york.ac.uk, identifier CRD42023409249.

## 1 Introduction

Diabetic kidney disease (DKD) is a microvascular complication caused by diabetes, which occurs in more than 40% of diabetic patients and is the leading reason for kidney failure worldwide (1). The prevalence of DKD is increasing due to the lack of early diagnosis and effective interventions (2). Renal biopsy is the gold standard for diagnosing kidney disease. Still, this method has limitations, such as being invasive and having sampling errors, so it is only used for a small percentage of patients with unclear diagnoses. Most clinical guidelines use the results of blood and urine biochemical markers as the preferred method for diagnosing and evaluating DKD (3). However, there are some inaccuracies in these blood and urine biochemical markers from the several clinical phenotypes that have been confirmed (4). This requires new, reproducible, non-invasive biomarkers to assess renal pathology in DKD.

Functional magnetic resonance imaging (fMRI) has shown great potential in assessing renal pathology in DKD. It is a non-invasive way to obtain more kidney structure and function biomarkers without exogenous contrast agents (5). Compared to kidney biopsy and serum or urine bioinformatic markers, fMRI has unique advantages (6).

Nowadays, the main fMRIs that have been used for structural or functional assessment of the kidney in diabetic kidney disease include magnetic resonance elastography (MRE-MRI), arterial spin labeling imaging (ASL-MRI), blood oxygen level-dependent imaging (BOLD-MRI), dilated weighted tensor imaging (DWI-MRI). MRE-MRI is a method to assess the degree of kidney elasticity and fibrosis by obtaining changes in tissue stiffness through shear waves generated by external vibrations with different conduction speeds in tissues of varying stiffness (7); ASL-MRI uses blood in the body as a contrast agent to track water protons in the blood and obtain tissue perfusion as a way to determine the progression and prognosis of diabetic kidney disease (8); BOLD-MRI shares similarities with ASL-MRI, except that BOLD-MRI uses deoxyhemoglobin to assess tissue oxygenation levels and assess renal hypoxia (9); DWI-MRI is an imaging that uses the diffusion of water molecules *in vivo* without contrast injection to assess microscopic changes in the kidney by mapping the movement of water within the tissue (10), Meanwhile, based on DWI-MRI theory, DTI-MRI and IVIM-MRI were born to address the deficiencies of DWI-MRI in microscopic orientation, distribution, diffusion and microperfusion effects of water molecules, and they enriched the acquisition of bioinformation markers (11). We summarize the advantages, disadvantages, and main parameters of fMRIs, which are currently used to assess DKD. Detailed information can be found in **Table 1**.

**Table 1:** Advantages and disadvantages of fMRI.

fMRI	Advantages	Disadvantages	Parameters
MRE-MRI	Virtual palpation; High reproducibility; Good stability	Decreased renal blood flow and tissue edema may lead to decreased liver stiffness; Shear wave propagation direction is unpredictable; Low anatomical resolution; Weight of artifact	Shear stiffness
ASL-MRI	Organ perfusion imaging without contrast media or invasive methods; Local blood flow can be examined	Low signal-to-noise ratio (SNR); Low spatial resolution; The renal medullary blood flow could not be evaluated; Lack of uniform standards	Renal blood flow (RBF)
BOLD-MRI	More mature; Directly reflects blood flow, blood volume, and oxidative metabolism; Distinguishing the renal cortex and medulla	Susceptible to interference by breathing or advocacy artifacts; Blood pH, temperature, and red blood cell volume can affect the partial pressure of oxygen in tissues; The relationship between blood flow and tissue uptake could not be clarified	Apparent relaxation rat (R2*)
DWI-MRI	The SNR was reduced by adjusting the b value	Breathing will generate artifacts; Hemorrhagic lesions may affect the imaging results; Two-dimensional space	Apparent diffusion coefficient (ADC)
DTI-MRI	Reflecting the movement of water molecules in multiple directions; Three-dimensional space	Capillary blood flow causes attenuation of the diffusion signal of water molecules	Fractional anisotropy (FA)
IVIM-MRI	Separate identification of microcirculation and true diffusion of water molecules; Breathe freely	Large variation in results for different b values; Intestinal motility can affect imaging	Perfusion fraction (f); True diffusion coefficient (D); Pseudo-diffusion coefficient (D*)

*In summary, noninvasive examination, no radiation, and no contrast agent are the common advantages of functional magnetic resonance imaging. For some patients with metal implants or claustrophobia, the choice of MRI technology is not timely, and the high cost is also a disadvantage of MRI.

The use of fMRI to assess renal structural and functional alterations in DKD is not yet widely available in the clinic, and relevant systematic reviews are limited. To investigate the value of functional magnetic resonance imaging for assessing renal structure and function in diabetic kidney disease, we performed this meta-analysis of several fMRIs.

## 2 Materials and methods

This Meta-Analysis was guided and performed by the Preferred Reporting Items for Systematic Reviews and Meta-Analysis (PRISMA) guidelines (12) and Meta-analysis Of Observational Studies in Epidemiology(MOOSE) (13, 14). A prospective protocol was developed and registered with PROSPERO (https://www.crd.york.ac.uk) under (ID: CRD42023409249). Informed consent was obtained from all authors for all included studies, and ethics committee approval was not required for further evaluation of published articles.

### 2.1 Data sources and search strategy

We searched PubMed, Web of Science, Cochrane Library, and Embase databases using a combination of medical subject headings (MeSH) and free words with Diabetic Nephropathies, Magnetic Resonance Imaging as the subject headings, and the specific search formula can be found in **Supplementary Materials Table S1**. The search time limit is from database creation to March 10, 2023.

### 2.2 Study selection and inclusion criteria

The inclusion criteria were as follows:

(1) Study subjects were DKD patients and healthy people;
(2) At least one functional magnetic resonance imaging technique is used;
(3) The mean (MN) and standard deviation (SD) of each parameter in the kidney cortex and medulla can be obtained;
(4) Full text available in Chinese or English.

The exclusion criteria were as follows:

(1) Overviews, conference papers, abstracts, reviews, and case reports;
(2) Basic research such as animal experiments;
(3) Literature with no access to data or data conversion;
(4) Literature with duplicate data;

### 2.3 Data extraction and assessment of the quality

After importing the searched literature into EndNote20, duplicates were automatically excluded. Two authors (ZQ Zhang; Y Chen) then read through the titles and abstracts of the literature to filter the literature related to the topic based on the inclusion and exclusion criteria. The full text was read to eliminate the literature that did not meet the requirements. Two authors (ZQ Zhang; S Liu) independently used Excel software to extract data, which mainly included literature title, author, year of publication, baseline information of patients, sample size, functional MRI information, eGFR formula, region of interests (ROIs), and MRI detection parameters. We used the Newcastle-Ottawa Scale (NOS) (15) to assess the quality of the included literature in terms of selected population, comparability of groups, and assessment of either the exposure or outcome of interest for case-control or cohort studies were scored on three dimensions. 1-3 were classified as low quality, 4-6 as moderate quality, and 7-9 as high quality. If there is a difference in the outcome, a third senior author(XQ Zhou) will be requested to make a judgment and final decision.

### 2.4 Statistical analysis

**Figure 1:**
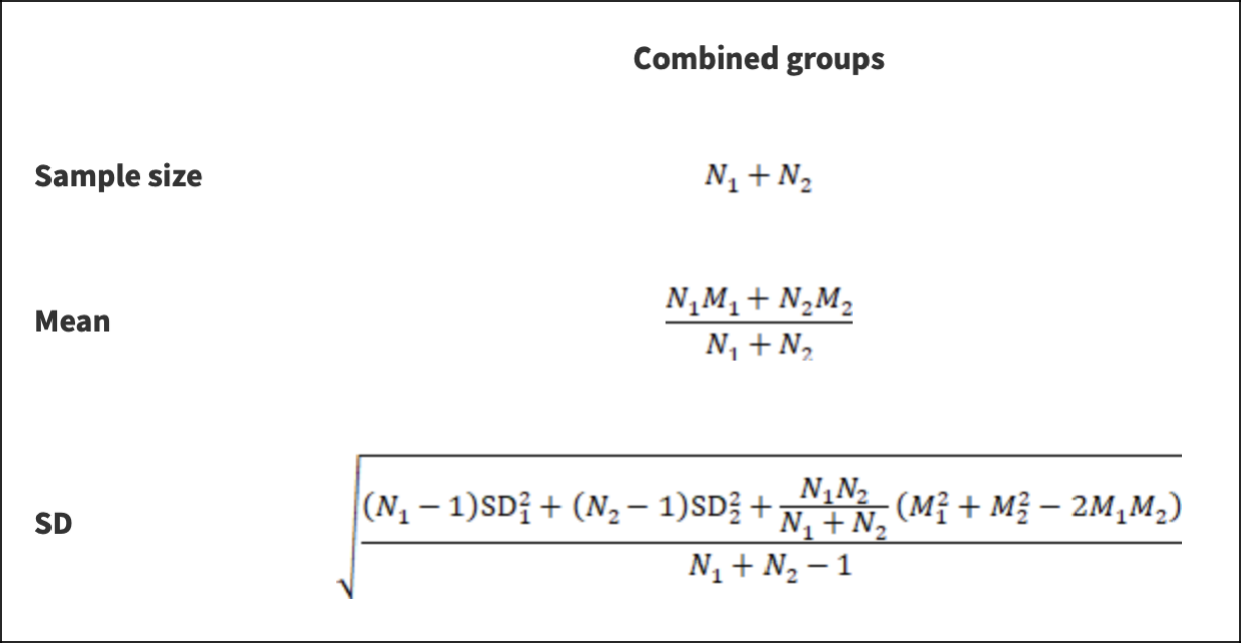
Formula of calculation. *N stands for sample size; M stands for the mean; SD stands for standard deviation; 1 and 2 stand for the two subgroups to be combined or the left and right kidney data of the same subset.

We combined the fMRI parameters in the literature for the right and left kidneys or for different CKD stages to have uniform criteria. The calculation formula (16) is shown in **Figure 1**. At the same time, we used an online transformation tool (https://www.math.hkbu.edu.hk/~tongt/papers/median2mean.html) to estimate the sample mean and standard deviation from the sample size, median, range and/or interquartile range (17, 18). Each parameter was analyzed by expressing the mean ± standard deviation and calculating the weighted mean difference (WMD) and 95% confidence interval (CI) in Review Manage 5.4 (Review Manager (RevMan) [Computer program]. Version 5.4.1, The Cochrane Collaboration, 2020.). We evaluated the heterogeneity of the individual studies by calculating the inconsistency index (I-squared, *I²*) statistics and cardinality test p-value (*p*) and selected fixed-effect or random-effect models based on the results. If the heterogeneity test result *I²*≤50% and *p*≥0.1, the fixed-effects model was used for data merging analysis. If the heterogeneity test result *I²*>50% and *p*<0.1, the random-effects model was used for merging analysis (19). If that measurement unit of a continuous variable is different, the unit conversion is carried out first; if the units of measure are the same, select the weighted mean difference for the subsequent analysis. The combined statistic was considered statistically significant at *P*<0. 05, and all effect sizes were expressed with 95% confidence intervals (95% CI). The sensitivity of this meta-analysis was assessed by selecting the manual one-by-one literature exclusion method to observe the changes in the combined results after excluding a particular literature. Egger’s test was performed separately on the included literature using Stata 16.0 to assess publication bias. An inverted symmetric funnel plot with *P* > 0.05 was considered evidence of slight publication bias.

## 3 Result

### 3.1 Search results

**Figure 2:**
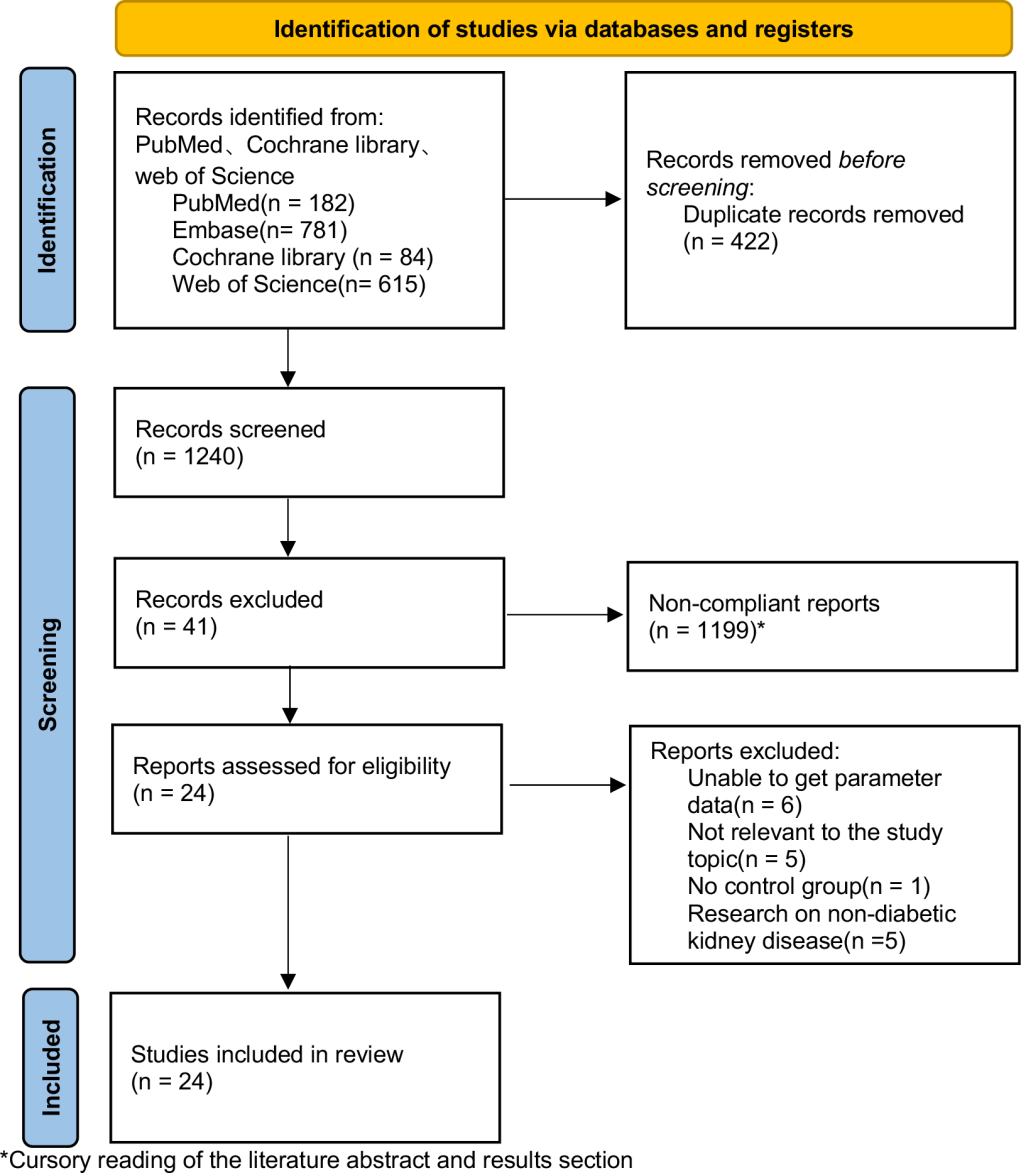
Characteristics of the included studies.

A total of 1240 articles were searched in PubMed, Embase, Web of Science, and Cochrane Library, and 24 papers were finally included, all of which were RCTs. The screening procedure is shown in **Figure 2**.

### 3.2 Eligible studies and patient characteristics

A total of 1565 study subjects were included in this study, including 1081 patients with DKD (269 patients with diabetes mellitus not combined with DKD), and 484 healthy volunteers in the control group. All of them were adults. The literature was published from 2011 to 2022. Twelve studies reported fMRI parameters in patients with diabetes mellitus not combined with DKD (20–31), and 9 studies were conducted on early diabetic kidney disease (21–24, 26, 29, 31–33). All included studies contained 6 kinds of fMRI. Among them, MRE was not counted in our follow-up study because the parametric results of only one study could not be pooled together for meta-analysis. Of these 24 papers, 5 studies used the ASL (20, 25, 34–36), 8 studies used BOLD (24, 26, 30, 34, 35, 37–39), 15 studies performed DTI (21–24, 27–30, 33, 35, 38, 40–43), and 5 studies used IVIM (21, 23, 31, 32, 35). Specific information on all included study literature is detailed in **Table 2**.

**Table 2:** Characteristics of the included studies.

Author, Year	fMRI information				Subject Information						Equation of eGFR
	Tesla	MR scaner	Types of fMRI	ROIs disposition	DKD	Health Volunteers	Non-DKD	Types of diabetes	Mean age of DKD	Mean age of HV	
Brown 2020 (20)	1.5T	GE Healthcare	ASL	NA	30	13	2	NA	60.2±14.2	50±17	CKD-EPI
Cakmak 2014 (40)	1.5T	GE Healthcare	DWI	More than 3 sites of interest on each renal cortex	78	22	0	NA	NA	NA	NA
Chen 2014 (22)	1.5T	Philips	DWI+DTI	More than 3 sites of interest on each renal parenchyma	30	12	14	T2DM	57±8	57±6	NA
Chen 2018 (21)	3.0T	Siemens	IVIM+DTI	NA	52	27	32	T2DM	61±8	59±7	MDRD
Deng 2018 (32)	1.5T	Siemens	IVIM	3 renal parenchymal sites of interest in each of the upper, middle and lower parts, avoiding the renal sinuses	19	12	0	T2DM	52.3	50.2	CKD-EPI
Feng 2018 (23)	3.0T	GE Healthcare	IVIM	1 site of interest in each of the bilateral upper, middle and lower renal parenchyma	40	20	20	T2DM	53.03±10.44	54.3±6.88	NA
Feng 2020 (24)	3.0T	GE Healthcare	DTI+BOLD	1 site of interest in each of the right kidney's superior and middle and inferior renal medulla	30	15	15	T2DM	54.17±10.83	50.8±8.05	MDRD
Jiang 2015 (37)	3.0T	Siemens	BOLD	1 site of interest in each of the bilateral upper, middle , lower renal cortex and medulla	34	11	0	NA	58	31	MDRD
Laursen 2022 (34)	3.0T	Philips	ASL+BOLD	NA	15	15	0	T1DM	58±14	56±15	NA
Liu 2017 (25)	3.0T	Siemens	ASL	3 sites of interest in each of the upper, middle and lower cortical areas bilaterally	50	25	25	T2DM	58.3±10.85	55.1±7.7	MDRD
Lu 2011 (41)	1.5T	Siemens	DTI	4 areas of interest in each of the bilateral kidneys near the hilum	16	5	0	NA	57±6	48±6	CKD-EPI
Makvandi 2022 (35)	NA	NA	ASL+BOLD+IVIM	NA	36	20	0	T2DM	68.6±5.6	66.7±6.2	CKD-EPI
Min 2021 (26)	3.0T	GE Healthcare	BOLD	3 sites of interest in the bilateral renal cortex and medulla upper, middle and lower medulla	58	30	30	T2DM	57.8±13.7	37.5±12.2	NA
Mohamed Osman 2021 (42)	1.5T	Philips	DWI	1 site of interest in each of the upper, middle and lower poles of the bilateral renal parenchyma	40	20	0	NA	51.2	40.05	CKD-EPI
Mora-Gutiérrez 2017 (36)	3.0T	Siemens	ASL	1 site of interest in each bilateral kidney	44	45	0	T2DM	67.02±9.27	59.76±10.02	MDRD/CKD-EPI
Mrđanin 2021 (43)^(p2)^	1.5T	GE Healthcare	DTI	6 sites of interest in each of the bilateral renal cortex and medulla	91	10	0	T2DM	62±9	32±5	CKD-EPI
Panduranga 2022 (27)	1.5T	Philips	DTI	1 site of interest in each of the upper, middle and lower poles of both kidneys	73	27	19	T2DM	53	NA	CKD-EPI
Saini 2018 (28)	1.5T	Philips	DTI	3 sites of interest in each of the bilateral renal cortex and medulla	83	30	23	NA	61	NA	CKD-EPI
Seah 2022 (38)	3.0T	Siemens	BOLD	3 sites of interest in each of the bilateral renal cortex and medulla	32	10	0	T1DM	45	45	CKD-EPI
Wang 2011 (39)	1.5T	GE Healthcare	BOLD	5-7 sites of interest in each of the bilateral renal cortex and medulla	20	7	0	T2DM	65	35	CKD-EPI
Wang 2018 (29)	3.0T	Siemens	DTI	1 sites of interest in each of the bilateral renal cortex and medulla	61	34	40	T2DM	61	59	MDRD
Wei 2022 (30)	3.0T	GE Healthcare	BOLD+DTI	1 sites of interest in each of the bilateral renal cortex and medulla	72	20	22	T2DM	57.3±14.05	59.3±10.1	MDRD
Ye 2019 (33 **)**	3.0T	GE Healthcare	DTI	1 site of interest in each of the anterior, middle and posterior aspects of the renal cortex and medulla bilaterally, near the renal pelvis.	36	26	0	T2DM	51±15	43±7	CKD-EPI
Zhang 2022 (31)	3.0T	Siemens	IVIM+DTI	1 sites of interest in each of the bilateral renal cortex and medulla	41	28	27	T2DM	47.14±10.01	49,89±10.8	CKD-EPI

The data were expressed as means ± standard deviation. CKD-EPI, Chronic Kidney Disease Epidemiology Collaboration equation; MDRD, Modification of Diet in Renal Disease equation; NA, Not Available.

### 33 Assessment of study quality

The quality of the included literature was assessed according to the NOS, which is shown in detail in **Supplementary Materials Figure S2**. According to the results, all studies were of high quality.

### 3.4 Meta-analysis results of 5 different types of fMRI

**Figure 3:**
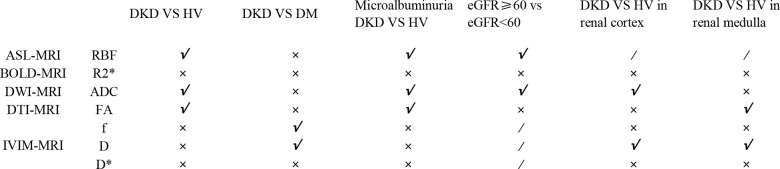
List of positive results of Meta-analysis.

A summary table of all positive results was drawn up for the reader to read quickly. The details can be found in the List of positive effects of Meta-analysis (**Figure 3**).

#### 3.4.1 ASL

ASL cannot detect renal medullary blood flow (44), as a result, comparing the renal cortex or medulla separately in the DKD and HV populations is impossible.

**Figure 4:**
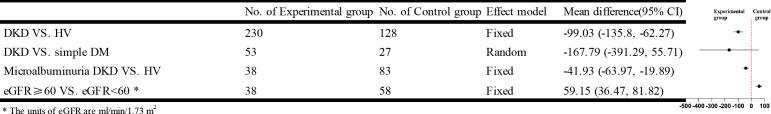
Forest plots for the RBF values. *The units of eGFR are ml/min/1.73m^2^.

Five studies using ASL-MRI compared DKD and HV. Statistical heterogeneity [*I²*=79%, *P*=0.0008] was observed between the studies after combined analysis, and Meta-analysis was performed using a random effects model. Meta-analysis showed that the renal blood flow (RBF) values were significantly lower in the DKD group than in the HV group, and the difference was statistically significant [WMD=-99.03, 95% CI (-135.8,-62.27), *P*<0.00001]. Two studies compared the DM group with DKD in non-diabetic kidney disease. But the difference was not statistically significant [WMD=-167.79, 95% CI (-391.29,-55.71), *P*<0.00001]. Three studies compared the RBF values of DKD and HV during microalbuminuria, and there was no statistical heterogeneity between studies after combined analysis [*I²*=0%, *P*=0.56]. Therefore, we used a fixed-effects model for Meta-analysis. The results showed that the RBF values of patients in the microalbuminuric DKD group were significantly lower than those in the HV group, and the difference was statistically significant [WMD=-41.93, 95% CI (-63.97,-19.89), *P*=0.0002]. Three studies analyzed the relationship between eGFR and RBF values. After combined analysis, there was no statistical heterogeneity between studies [*I²*=51%, *P*=0.13], so we used a fixed-effects model for Meta-analysis. Meta-analysis showed that patients in the DKD group with the estimated glomerular filtration rate (eGFR) ≥ 60 ml/min/1.73 m^2^ had significantly higher RBF values than patients in the DKD group with eGFR < 60 ml/min/1.73 m^2^, and the difference was statistically significant [WMD=59.15, 95% CI (36.47,81.82), *P*=0.0005]. The brief forest plots can be found in **Figure 4**. Detailed data can be found in **Supplementary Materials S5**.

#### 3.4.2 BOLD

**Figure 5:**
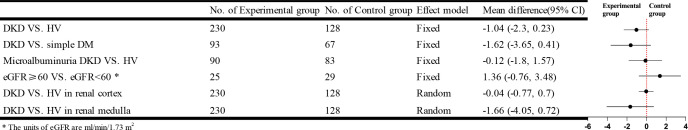
Forest plots for the R2* values.

Eight studies that used BOLD-MRI compared DKD with HV while three studies compared the simple DM group with DKD, five studies compared BOLD parameters of microalbuminuric DKD with HV, and two studies analyzed the relationship between eGFR and the apparent relaxation rat (R2*) values, but the difference was not statistically significant. The brief forest plots can be found in **Figure 5**. Detailed data can be found in **Supplementary Materials S5**.

#### 3.4.3 DTI-DWI

**Figure 6:**
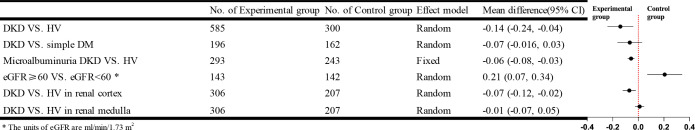
Forest plots for the ADC values.

Twelve studies that used DTI-MRI and three studies that used DWI-MRI compared DKD with HV, and there was statistical heterogeneity between studies after combined analysis [*I²*=93%, *P*<0.00001]. Meta-analysis of the random-effects model showed that patients in the DKD group had significantly lower apparent diffusion coefficient (ADC) values than the HV group, and the difference was statistically significant [WMD=-0.14, 95% CI (-0.24,-0.04), *P*=0.005]. Ten studies compared the ADC values in cortical or medullary DKD and HV groups, respectively, with statistical heterogeneity between studies after combined analysis [*I²*=64%, *P*=0.003]; [*I²*=71%, *P*=0.0002]. We used a random effects model for meta-analysis. The results showed that patients in the DKD group had significantly lower cortical ADC values than the HV group, and the difference was statistically significant [WMD=-0.07, 95%CI (-0.12,-0.02), *P*=0.009]; while the comparison of the renal medulla alone was not significantly different and not statistically significant [WMD=-0.01, 95%CI (-0.07,0.05), *P*=0.72]. Seven studies compared the simple DM group with the DKD group, and there was statistical heterogeneity between studies after combined analysis [*I²*=71%, *P*=0.002]. Meta-analysis using a random effects model showed that the ADC values were lower in the DKD group than in the simple DM group. Still, the difference was insignificant [WMD=-0.07, 95% CI (-0.16,0.03), *P*=0.16]. Twelve studies compared the results of the ADC values between DKD in microalbuminuria and HV. After combined analysis, there was no statistical heterogeneity between studies [*I²*=48%, *P*=0.03], and Meta-analysis was performed using a fixed effects model. The results showed that patients in the DKD group with microalbuminuria had lower ADC values than the HV group, and the difference was statistically significant [WMD=-0.06, 95% CI (-0.08,-0.03), *P*<0.0001]. Five studies analyzed the relationship between eGFR and ADC values, and after combined analysis, there was statistical heterogeneity between studies [*I²*=87%, *P*<0.00001]. Meta-analysis using a random-effects model showed that patients in the DKD group with eGFR ≥ 60 ml/min/1.73 m^2^ had higher ADC values than patients in the DKD group with eGFR < 60 ml/min/1.73 m^2^. This difference was statistically significant [WMD=0.21, 95% CI (0.07,0.34), *P*=0.002]. The brief forest plots can be found in **Figure 6**. Detailed data can be found in **Supplementary Materials S5**.

**Figure 7:**
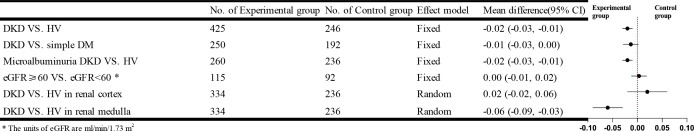
Forest plots for the FA values. *The units of eGFR are ml/min/1.73.m^2^.

Twelve studies that used DTI-MRI compared DKD and HV, and after combined analysis, there was no statistical heterogeneity between studies [*I²*=32%, *P*=0.13]. Meta-analysis was performed using a fixed-effects model, and the results of the Meta-analysis showed that the fractional anisotropy (FA) values of patients in the DKD group were significantly lower than those in the HV group. The difference was statistically significant [WMD=-0.02, 95% CI (-0.03,-0.01), *P*<0.0001]. Eleven studies targeted the renal cortex or the renal medulla, comparing the DKD and HV groups. There was statistical heterogeneity between studies after combined analysis [*I²*=97%, *P*<0.00001]; [*I²*=94%, *P*<0.00001]. A random effects model was used to analyze the included studies, and there was no significant difference between the two groups in the renal cortex [WMD=0.02, 95%CI (-0.02,0.06), *P*=0.31]; comparison from the renal medulla alone showed that patients in the DKD group had significantly lower medullary FA values than the HV group and the difference was statistically significant [WMD= -0.06, 95% CI (-0.09,-0.03), *P*<0.00001]. Eight studies compared the simple DM group with the DKD group. The results of the Meta-analysis showed that the difference was not statistically significant. Eleven studies compared the FA values of microalbuminuria DKD with HV. After combined analysis, there was no statistical heterogeneity between studies [*I²*=16%, *P*=0.29]. Meta-analysis was performed using a fixed effects model. Meta-analysis results showed that patients in the microalbuminuric DKD group had lower FA values than the HV group, with a statistically significant difference [WMD=-0.02, 95% CI (-0.03,-0.01), *P*<0.0001]. Four studies analyzed the relationship between eGFR and the FA values, and after combined analysis, the FA values of patients in the DKD group with eGFR<60 ml/min/1.73 m^2^ were not significantly different from those of patients in the DKD group with eGFR≥60 ml/min/1.73 m^2^. The brief forest plots can be found in **Figure 7**. Detailed data can be found in **Supplementary Materials S5**.

#### 3.4.4 IVIM-DWI

The study trial groups using this fMRI technique were all patients with DKD in the microalbuminuric stage, so there were not enough data for subgroup analysis according to eGFR staging.

**Figure 8:**
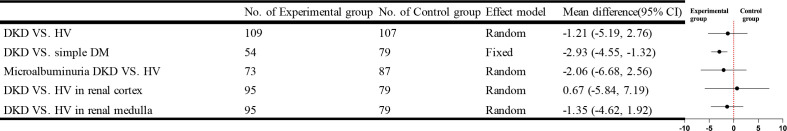
Forest plots for the f values.

Five studies that used IVIM compared DKD with HV, and the result showed that patients in the DKD group had lower perfusion fraction (f) values than the HV group, but the difference was not statistically significant. Four studies were recorded separately from the renal cortex and renal medulla, and no statistically significant cortical or medullary f values compared the DKD group with the HV group. Three studies compared the simple DM group with DKD, and the combined analysis revealed no statistical heterogeneity between studies [*I²*=24%, *P*=0.27]. Meta-analysis was performed using a fixed effects model. The results of the Meta-analysis showed that the DKD group had lower f values than the DM group, and the difference was statistically significant [WMD=-2.93, 95% CI (-4.55,-1.32), *P*=0.0004]. Four studies compared f values in microalbuminuric DKD with HV, and after combined analysis, there was no statistically significant. The brief forest plots can be found in **Figure 8**. Detailed data can be found in **Supplementary Materials S5**.

**Figure 9:**
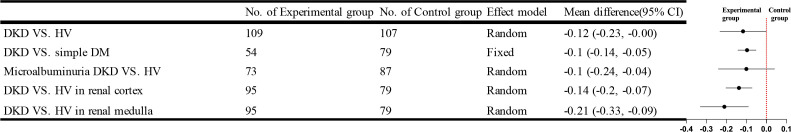
Forest plots for the D values.

Five studies using IVIM compared DKD and HV. The results showed no statistically significant difference between the true diffusion coefficient (D) values of the DKD group and those of the HV group. Four studies compared cortical or medullary D values of the DKD group and the HV group, respectively, and there was statistical heterogeneity among the studies [*I²*=57%, *P*=0.07]; [*I²*=84%, *P*=0.0003]. A random effects model was used for comparison. The result showed that cortical or medullary D values in the DKD group were lower than those in the HV group, and both were statistically significant [WMD=-0.14, 95%CI (-0.20,-0.07), *P*<0.0001]; [WMD=-0.21, 95%CI (-0.33,-0.09), *P*=0.0004]. Three studies compared the differences between the simple DM and DKD groups, and there was no statistical heterogeneity among the studies after combined analysis [*I²*=0%, *P*=0.81]. Meta-analysis using a fixed effect model showed that the D values of the DKD group were lower than that of the DM group, and the difference was statistically significant [WMD=-0.10, 95%CI (-0.14,-0.05), *P*<0.0001]. Four studies compared the parameter D values of the microalbuminuria DKD group and HV group. After the combined analysis, there was no statistically significant [WMD=-0.10, 95%CI (-0.24,0.04), *P*=0.17]. The brief forest plots can be found in **Figure 9**. Detailed data can be found in **Supplementary Materials S5**.

**Figure 10:**
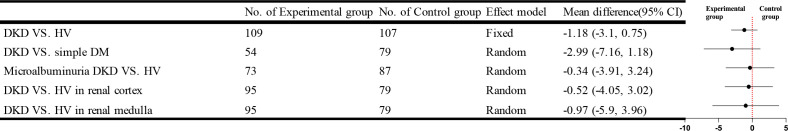
Forest plots for the D* values.

Five studies using IVIM compared DKD and HV, four studies recorded the results from the renal cortex and medulla separately, three studies compared the difference in the pseudo-diffusion coefficient (D*) values between the simple DM and DKD groups and four studies compared the D^*^ values of microalbuminuria DKD and HV, no statistical differences were found in any of them. The brief forest plots can be found in **Figure 10**. Detailed data can be found in **Supplementary Materials S5**.

### 3.5 Sensitivity analysis and publication bias analysis

After excluding one piece of literature in the meta-analysis one by one, we did not find any change in the results. Therefore, the positive effects of this meta-analysis are stable and reliable. Egger’s test of positive results showed that there was no publication bias, and the specific results can be found in **Supplementary Materials Figure S3**.

## 4 Discussion

Nowadays, more and more studies have shown many different phenotypes for the progression of DKD. For phenotypes other than the classical phenotype, using eGFR with proteinuria to assess DKD no longer seems appropriate (4). Although renal puncture biopsy can avoid such issues, it is not absolute. In the early stages of DKD, atypical biopsy findings may be present. In contrast, fMRI is cheaper, less painful, and more acceptable to patients than a renal biopsy and can avoid the risk of sampling errors. While compared to serum and urine biomarkers, patients’ results are less subject to external influences, with more biomarker information and a more comprehensive and detailed assessment of the kidney. Despite its drawbacks, fMRI remains a promising non-invasive, contrast agent-free method of detecting kidney morphology or function.

Many studies have shown that fMRI has unique advantages in detecting and differentiating chronic kidney disease (CKD). Zeng et al. (45) found that renal cortical RBF, f, D, and D* parameters were significantly and positively correlated with eGFR in patients with chronic kidney disease using ASL and IVIM, and the f values even exceeded eGFR in distinguishing CKD from HV on the areas under the curve (AUC). Qin et al. (46) performed a meta-analysis on the effectiveness of fMRI for identifying early CKD. It concluded that BOLD, DTI, and IVIM could distinguish the early CKD population from the general population and that DWI has an advantage in diagnosing all stages of CKD. Niu et al. (47) summarized studies using DWI to assess various stages of CKD and concluded that DWI could be used for early diagnosis and staging of CKD. DKD is a type of CKD that is heterogeneous from other types of CKD, so a meta-analysis is necessary to address the value of multiple fMRIs in assessing renal injury in DKD.

This study shows promising results for ASL in distinguishing DKD from healthy people and patients with early and moderately advanced DKD. This suggests that the RBF values are negatively correlated with the course of DKD in patients. However, it does not seem to distinguish diabetic patients from those with diabetic kidney disease. Such a result may be related to the number of included literature or the fact that high glucose toxicity at the beginning of diabetic disease already mediates multiple homologous pathways causing glomerular basement membrane thickening (48). Unfortunately, we could not search to find studies in this area. The R2* values in the meta-analysis of BOLD did not show its usefulness for assessing DKD. Previous studies have shown that BOLD is of limited importance in responding to long-term renal fibrosis (49). Michaely et al. (50) also confirmed that R2* did not differ significantly in different stages of CKD, including DKD. We make the speculation that arterio-venous oxygen shunting facilitates the dynamic regulation of renal oxygenation (51), making renal hypoxia less pronounced in the course of chronic kidney disease. Therefore, BOLD may help diagnose acute kidney injury but is insignificant in assessing chronic kidney disease.

The role of DTI-DWI in the assessment of DKD disease is satisfactory. We found a significant difference in renal cortical ADC values between DKD patients and healthy volunteers. However, the medullary ADC values cannot distinguish DKD from HV nor determine DKD in microalbuminuria from simple DM. We also found lower ADC values in early DKD compared to mid to late-stage DKD. And the FA values seemed to complement the ADC values because the FA parameters differed significantly between DKD and HV patients in the renal medulla. However, it cannot distinguish between the FA values in patients with DKD and simple DM or early DKD and intermediate to late DKD. The physiology of the kidney determines such a result, as the renal tubules and collecting ducts enter the renal pelvis in a radial pattern (52). The main difference between DTI and DWI is the introduction of three-dimensional space. DTI can respond to the axial or longitudinal diffusion rate of water molecules, so DTI is more significant in responding to the renal medulla. In contrast, DWI can be susceptible to blood perfusion (53). Compared to the renal medulla, the renal cortex requires more blood perfusion to perform the physiological function of filtering blood. Therefore, DWI and DTI must complement each other to assess the extent of renal parenchymal injury in DKD.

IVIM-DWI uses a bi-exponential model to enhance DWI’s perception of capillary perfusion and diffusion, making it more accurate in assessing renal function or microstructure (54). Compared to the D value, the f and D^*^ values are limited in determining DKD. We found significant differences in cortical, medullary, or parenchyma D values between the DKD and HV groups and differences in D values between the DKD and simple DM groups. In contrast, the f values were significantly different only in comparing the DKD and simple diabetes groups. No significant differences in the D^*^ values were found in several group comparisons. The results of this study are consistent with the study of Ren et al. (31). The difference in f-values between the DKD group and the simple DM group demonstrated their sensitivity in assessing pathological changes in DKD, which was also confirmed in the study by Qin et al. (46). The D* and f values reflect the microcirculatory perfusion and the D value demonstrates the movement of water molecules (55), so we speculate that such results are related to the included studies being all for early DKD. Although there is microcirculatory damage in early DKD, the reliable compensatory mechanism of the kidney allows for faster blood flow, evidenced by the altered hemodynamics in DKD, thus compensating for the lack of f and D* values. Schurek et al. (56) showed no effect of these pathological alterations on the diffusion of water molecules, which does not agree with our findings, as we found statistical differences in the distribution of water molecules between early DKD and healthy populations. Therefore, we would like more studies to focus on the differences in IVIM parameters between early DKD and mid to late-stage DKD as a way to verify our conjecture.

In the course of DKD, renal hemodynamic changes, hypoxia, and even glomerulosclerosis or interstitial fibrosis (4), can be reflected in functional magnetic resonance imaging parameters. With so many types of fMRI, the pathological changes they reflect have their preferences. ASL and BOLD were mainly used to assess renal perfusion; DWI, DTI, and IVIM were used to evaluate the diffusion of water molecules in the kidney. Therefore, Makvandi *et al. (* 35) combined multiple fMRI analyses, which seems more accurate than a single fMRI for assessing pathological changes in DKD. Such an idea has also been promoted by Mehmet et al. (53). We believe combining multiple fMRI test parameters is necessary to assess the renal structure and function and establish uniform and standardized test criteria. In addition, uniform standards for parameter ranges need to be installed, as is the case for blood and urine biochemical markers. Only when these consistent criteria are established it will be possible to quantify the assessment of structural or functional changes in the kidney by fMRI.

In addition to the several types of fMRI included in this paper, there are several other functional magnetic resonance imaging. For example, magnetic resonance spectroscopy imaging (MRS-MRI) and magnetic susceptibility weighted imaging (SWI-MRI). MRS-MRI is mainly used to determine the concentration of compounds and metabolites in tissues, and is currently primarily used in brain tissue-related studies and has not been widely used in diabetic kidney disease (57); SWI-MRI is a further development of the BOLD-MRI technique, which uses the different magnetic susceptibility of tissues to perform imaging by identifying high concentrations of deoxygenated hemoglobin in the veins in contrast to surrounding tissues and is more suitable for the identification of hemorrhagic disorders. However, the use of SWI-DWI is controversial owing to the unique physiological characteristics of the renal body (58). Due to the limitations of the number of included studies, a meta-analysis of these types of fMRI could not be performed. More clinical studies are needed in the future to enrich our conclusions.

This study has the following shortcomings: 1. The included literature is only in English, which may have a particular bias; 2. Different measurement methods, MRI scanners, field strengths, and standards for determining ROIs heterogeneity cannot be ruled out; 3. All the included studies were not diagnosed by renal puncture, so there was a deviation in the diagnosis of DKD; 4. We did not collect data on how DKD compares to other CKD, which is essential for making a differential diagnosis clinically. To address these shortcomings, we hope they can be remedied in the subsequent study.

Functional MRI is not necessary for the diagnosis of DKD. Therefore, fMRI has not been widely promoted in the field of DKD, which may be due to its high price or the lack of uniform standards. Meanwhile, MRI requires more imaging time compared to X-rays. Any slight movement can have an impact on the quality of the image. It is also a problem for the same patient to have significantly different renal fMRI findings due to differences in diet or medications taken. These issues have been raised in the consensus (59, 60), but more clinical studies are needed to address them. Any new science and technology need to develop and grow with continuous exploration. Although functional magnetic resonance imaging is not mature, we believe that fMRI to evaluate DKD or other renal diseases can be widely respected and used.

In conclusion, ASL and DWI on parameters can better distinguish DKD patients and healthy people. The parameters RBF and ADC have certain values in distinguishing DKD staging. In the future, we hope that more investigators will see the value of fMRI in the assessment of DKD so that this contrast-free, noninvasive test will be more involved in clinical decision-making and prognosis determination.

## Data availability statement

The original contributions presented in the study are included in the article/**Supplementary Material**. Further inquiries can be directed to the corresponding author.

## Author contributions

ZZ designed the meta-analysis. ZZ, YC, and SL selected the eligible articles. ZZ and SL abstracted the data. ZZ and JY analyzed the data. ZZ and YC wrote the paper. ZZ, YC, XZ, SL; JY interpreted the results; and all authors approved submitting the final manuscript. All authors contributed to the article and approved the submitted version.
