# Corrigendum: The value of functional magnetic resonance imaging in the evaluation of diabetic kidney disease: a systematic review and meta-analysis

**DOI:** 10.3389/fendo.2023.1270152

**Published:** 2023-08-25

**Authors:** Ziqi Zhang, Yu Chen, Xiqiao Zhou, Su Liu, Jiangyi Yu

**Affiliations:** ^1^ Department of Endocrinology, Jiangsu Provincial Hospital of Chinese Medicine, Affiliated Hospital of Nanjing University of Chinese Medicine, Nanjing, Jiangsu, China; ^2^ The First Clinical Medical College of Nanjing University of Chinese Medicine, Nanjing, China

**Keywords:** functional magnetic resonance imaging, diabetic kidney disease, meta-analysis, fMRI, DKD

In the published article, there was an error in affiliation 1. Instead of “The First Clinical Medical College of Nanjing University of Chinese Medicine, Nanjing, China”, it should be “Department of Endocrinology, Jiangsu Provincial Hospital of Chinese Medicine, Affiliated Hospital of Nanjing University of Chinese Medicine, Nanjing, Jiangsu, China”.

In the published article, there was an error in affiliation 2. Instead of “Department of Endocrinology, Jiangsu Provincial Hospital of Traditional Chinese Medicine, Affiliated Hospital of Nanjing University of Traditional Chinese Medicine, Nanjing, Jiangsu Province, China”, it should be “The First Clinical Medical College of Nanjing University of Chinese Medicine, Nanjing, China”.

In the published article, there was an error regarding the affiliations for Ziqi Zhang and Yu Chen. As well as having affiliation *The First Clinical Medical College of Nanjing University of Chinese Medicine, Nanjing, China*, they should also have *Department of Endocrinology, Jiangsu Provincial Hospital of Chinese Medicine, Affiliated Hospital of Nanjing University of Chinese Medicine, Nanjing, Jiangsu, China*.

The authors apologize for these errors and state that these do not change the scientific conclusions of the article in any way. The original article has been updated.

