# Supplementary material for: The value of functional magnetic resonance imaging in the evaluation of diabetic kidney disease: a systematic review and meta-analysis
Source: Front Endocrinol (Lausanne). 2023 Jul 7;14:1226830. doi: 10.3389/fendo.2023.1226830 (PMC10360195; doi:10.3389/fendo.2023.1226830)
Supplement: Supplementary file 1 [file DataSheet_1.pdf]

Supplementary materials

Table S1. Search strategies

Database	Search strategy	Results	
1)PubMed (To March 10,2023)	#1	((((((((((((((("Diabetic Nephropathies"[Mesh]) OR (Nephropathies, Diabetic[Title/Abstract])) OR (Nephropathy, Diabetic[Title/Abstract])) OR (Diabetic Nephropathy[Title/Abstract])) OR (Diabetic Kidney Disease[Title/Abstract])) OR (Diabetic Kidney Diseases[Title/Abstract])) OR (Kidney Disease, Diabetic[Title/Abstract])) OR (Kidney Diseases, Diabetic[Title/Abstract])) OR (Diabetic Glomerulosclerosis[Title/Abstract])) OR (Glomerulosclerosis, Diabetic[Title/Abstract])) OR (Intracapillary Glomerulosclerosis[Title/Abstract])) OR (Nodular Glomerulosclerosis[Title/Abstract])) OR (Glomerulosclerosis, Nodular[Title/Abstract])) OR (Kimmelstiel-Wilson Syndrome[Title/Abstract])) OR (Kimmelstiel Wilson Syndrome[Title/Abstract])) OR (Syndrome, Kimmelstiel-Wilson[Title/Abstract])) OR (Kimmelstiel-Wilson Disease[Title/Abstract])) OR (Kimmelstiel Wilson Disease[Title/Abstract])	38957
	#2	((((((((((((((((((((((((((((((((((("Magnetic Resonance Imaging"[Mesh]) OR (Imaging, Magnetic Resonance[Title/Abstract])) OR (NMR Imaging[Title/Abstract])) OR (Imaging, NMR[Title/Abstract])) OR (Tomography, NMR[Title/Abstract])) OR (Tomography, MR[Title/Abstract])) OR (MR Tomography[Title/Abstract])) OR (NMR Tomography[Title/Abstract])) OR (Steady-State Free Precession MRI[Title/Abstract])) OR (Steady State Free Precession MRI[Title/Abstract])) OR (Zeugmatography[Title/Abstract])) OR (Imaging, Chemical Shift[Title/Abstract])) OR (Chemical Shift Imagings[Title/Abstract])) OR (Imagings, Chemical Shift[Title/Abstract])) OR (Shift Imaging, Chemical[Title/Abstract])) OR (Shift Imagings, Chemical[Title/Abstract])) OR (Chemical Shift Imaging[Title/Abstract])) OR (Magnetic Resonance Image[Title/Abstract])) OR (Image, Magnetic Resonance[Title/Abstract])) OR (Magnetic Resonance	561426

		Images[Title/Abstract])) OR (Resonance Image, Magnetic[Title/Abstract])) OR (Magnetization Transfer Contrast Imaging[Title/Abstract])) OR (MRI Scans[Title/Abstract])) OR (MRI Scan[Title/Abstract])) OR (Scan, MRI[Title/Abstract])) OR (Scans, MRI[Title/Abstract])) OR (Tomography, Proton Spin[Title/Abstract])) OR (Proton Spin Tomography[Title/Abstract])) OR (fMRI[Title/Abstract])) OR (MRI, Functional[Title/Abstract])) OR (Functional MRI[Title/Abstract])) OR (Functional MRIs[Title/Abstract])) OR (MRIs, Functional[Title/Abstract])) OR (Functional Magnetic Resonance Imaging[Title/Abstract])) OR (Magnetic Resonance Imaging, Functional[Title/Abstract])) OR (Spin Echo Imaging[Title/Abstract])) OR (Echo Imaging, Spin[Title/Abstract])) OR (Echo Imagings, Spin[Title/Abstract])) OR (Imaging, Spin Echo[Title/Abstract])) OR (Imagings, Spin Echo[Title/Abstract])) OR (Spin Echo Imagings[Title/Abstract])	
	#3	#1 AND #2	182
2) Embase (To March 10,2023)	#1	'diabetic nephropathies'/exp OR 'diabetic nephropathies' OR 'nephropathies, diabetic' OR 'nephropathy, diabetic'/exp OR 'nephropathy, diabetic' OR 'diabetic nephropathy'/exp OR 'diabetic nephropathy' OR 'diabetic kidney disease'/exp OR 'diabetic kidney disease' OR 'diabetic kidney diseases' OR 'kidney disease, diabetic' OR 'kidney diseases, diabetic' OR 'diabetic glomerulosclerosis'/exp OR 'diabetic glomerulosclerosis' OR 'glomerulosclerosis, diabetic'/exp OR 'glomerulosclerosis, diabetic' OR 'intracapillary glomerulosclerosis' OR 'nodular glomerulosclerosis'/exp OR 'nodular glomerulosclerosis' OR 'glomerulosclerosis, nodular' OR 'kimmelstiel-wilson syndrome'/exp OR 'kimmelstiel-wilson syndrome' OR 'kimmelstiel wilson syndrome'/exp OR 'kimmelstiel wilson syndrome' OR 'syndrome, kimmelstiel-wilson' OR 'kimmelstiel-wilson disease'/exp OR 'kimmelstiel-wilson disease' OR 'kimmelstiel wilson disease'/exp OR 'kimmelstiel wilson disease'	60503
	#2	'magnetic resonance imaging'/exp OR 'magnetic resonance imaging' OR 'imaging, magnetic resonance' OR 'nmr imaging or imaging, nmr' OR 'tomography, nmr or tomography, mr' OR 'mr tomography' OR 'nmr	1237031

		tomography' OR 'steady-state free precession mri' OR 'steady state free precession mri' OR 'zeugmatography' OR 'imaging, chemical shift' OR 'chemical shift imagings' OR 'imagings, chemical shift' OR 'shift imaging, chemical' OR 'shift imagings, chemical' OR 'chemical shift imaging'/exp OR 'chemical shift imaging' OR 'magnetic resonance image' OR 'image, magnetic resonance' OR 'magnetic resonance images' OR 'resonance image, magnetic' OR 'magnetization transfer contrast imaging' OR 'mri scans' OR 'mri scan' OR 'scan, mri' OR 'scans, mri' OR 'tomography, proton spin' OR 'proton spin tomography' OR 'fmri'/exp OR 'fmri' OR 'mri, functional' OR 'functional mri'/exp OR 'functional mri' OR 'functional mris' OR 'mris, functional' OR 'functional magnetic resonance imaging'/exp OR 'functional magnetic resonance imaging' OR 'magnetic resonance imaging, functional'/exp OR 'magnetic resonance imaging, functional' OR 'spin echo imaging'/exp OR 'spin echo imaging' OR 'echo imaging, spin' OR 'echo imagings, spin' OR 'imaging, spin echo' OR 'imagings, spin echo' OR 'spin echo imagings'	
	#3	#1 AND #2	781
3) Cochrane Library (To March 10,2023)	#1	MeSH descriptor: [Diabetic Nephropathies] explode all trees OR (Nephropathies, Diabetic OR Nephropathy, Diabetic OR Diabetic Nephropathy OR Diabetic Kidney Disease OR Diabetic Kidney Diseases OR Kidney Disease, Diabetic OR Kidney Diseases, Diabetic OR Diabetic Glomerulosclerosis OR Glomerulosclerosis, Diabetic OR Intracapillary Glomerulosclerosis OR Nodular Glomerulosclerosis OR Glomerulosclerosis, Nodular OR Kimmelstiel-Wilson Syndrome OR Kimmelstiel Wilson Syndrome OR Syndrome, Kimmelstiel-Wilson OR Kimmelstiel-Wilson Disease OR Kimmelstiel Wilson Disease):ti,ab,kw	6848
	#2	MeSH descriptor: [Magnetic Resonance Imaging] explode all trees OR (Imaging, Magnetic Resonance OR NMR Imaging OR Imaging, NMR OR Tomography, NMR OR Tomography, MR OR MR Tomography OR NMR Tomography OR Steady-State Free Precession MRI OR Steady State Free Precession MRI OR Zeugmatography OR Imaging, Chemical Shift OR Chemical Shift Imagings OR Imagings, Chemical Shift OR Shift Imaging, Chemical OR Shift Imagings, Chemical OR Chemical Shift Imaging OR	47669

		Magnetic Resonance Image OR Image, Magnetic Resonance OR Magnetic Resonance Images OR Resonance Image, Magnetic OR Magnetization Transfer Contrast Imaging OR MRI Scans OR MRI Scan OR Scan, MRI OR Scans, MRI OR Tomography, Proton Spin OR Proton Spin Tomography OR fMRI OR MRI, Functional OR Functional MRI OR Functional MRIs OR MRIs, Functional OR Functional Magnetic Resonance Imaging OR Magnetic Resonance Imaging, Functional OR Spin Echo Imaging OR Echo Imaging, Spin OR Echo Imagings, Spin OR Imaging, Spin Echo OR Imagings, Spin Echo OR Spin Echo Imagings):ti,ab,kw	
		#1 AND #2	84
4) Web of science (To March 10,2023)	#1	TS=(Diabetic Nephropathies OR Nephropathies, Diabetic OR Nephropathy, Diabetic OR Diabetic Nephropathy OR Diabetic Kidney Disease OR Diabetic Kidney Diseases OR Kidney Disease, Diabetic OR Kidney Diseases, Diabetic OR Diabetic Glomerulosclerosis OR Glomerulosclerosis, Diabetic OR Intracapillary Glomerulosclerosis OR Nodular Glomerulosclerosis OR Glomerulosclerosis, Nodular OR Kimmelstiel-Wilson Syndrome OR Kimmelstiel Wilson Syndrome OR Syndrome, Kimmelstiel-Wilson OR Kimmelstiel-Wilson Disease OR Kimmelstiel Wilson Disease)	82733
	#2	TS=(Magnetic Resonance Imaging OR Imaging, Magnetic Resonance OR NMR Imaging OR Imaging, NMR OR Tomography, NMR OR Tomography, MR OR MR Tomography OR NMR Tomography OR Steady-State Free Precession MRI OR Steady State Free Precession MRI OR Zeugmatography OR Imaging, Chemical Shift OR Chemical Shift Imagings OR Imagings, Chemical Shift OR Shift Imaging, Chemical OR Shift Imagings, Chemical OR Chemical Shift Imaging OR Magnetic Resonance Image OR Image, Magnetic Resonance OR Magnetic Resonance Images OR Resonance Image, Magnetic OR Magnetization Transfer Contrast Imaging OR MRI Scans OR MRI Scan OR Scan, MRI OR Scans, MRI OR Tomography, Proton Spin OR Proton Spin Tomography OR fMRI OR MRI, Functional OR Functional MRI OR Functional MRIs OR MRIs, Functional OR Functional Magnetic Resonance Imaging OR Magnetic Resonance Imaging, Functional OR Spin Echo Imaging OR Echo Imaging, Spin OR Echo Imagings, Spin OR Imaging, Spin Echo OR	915214

		Imagings, Spin Echo OR Spin Echo Imagings)	
	#3	#1 AND #2	615

Figure S2. Quality assessment of the included studies

	Selection				Comparability	Outcome			Scores
**Study**	Adequate definition of cases	Representativeness of the cases	Selection of controls	Definition of controls	Control for important factor	Ascertainment of outcome	Same method of ascertainment for cases and controls	Non-response rate	
Brown 2020	1	1	0	1	2	1	1	1	8
Cakmak 2014	1	1	0	1	1	1	1	1	7
Chen 2014	1	1	1	1	2	1	1	1	9
Chen 2018	1	1	1	1	2	1	1	1	9
Deng 2018	1	1	0	1	1	1	1	1	7
Feng 2018	1	1	1	1	2	1	1	1	9
Feng 2020	1	1	1	1	2	1	1	1	9
Jiang 2015	1	1	0	1	1	1	1	1	7
Laursen 2022	1	1	1	1	1	1	1	1	8
Liu 2017	1	1	0	1	2	1	1	1	8
Lu 2011	1	1	1	1	1	1	1	1	8
Makvandi 2022	1	1	1	1	1	1	1	1	8
Min 2021	1	1	0	1	2	1	1	1	8
Mohamed Osman 2021	1	1	1	1	1	1	1	1	8
Mora-Gutiérrez 2017	1	1	0	1	1	1	1	1	7
Mrđanin 2021	1	1	0	1	1	1	1	1	7
Panduranga 2022	1	1	0	1	2	1	1	1	8
Saini 2018	1	1	0	1	2	1	1	1	8
Seah 2022	1	1	1	1	1	1	1	1	8
Wang 2011	1	1	0	1	1	1	1	1	7
Wang 2018	1	1	1	1	2	1	1	1	9
Wei 2022	1	1	1	1	2	1	1	1	9
Ye 2019	1	1	1	1	1	1	1	1	8
Zhang 2022	1	1	0	1	2	1	1	1	8

Figure S3. Published risk of bias chart

RBF:

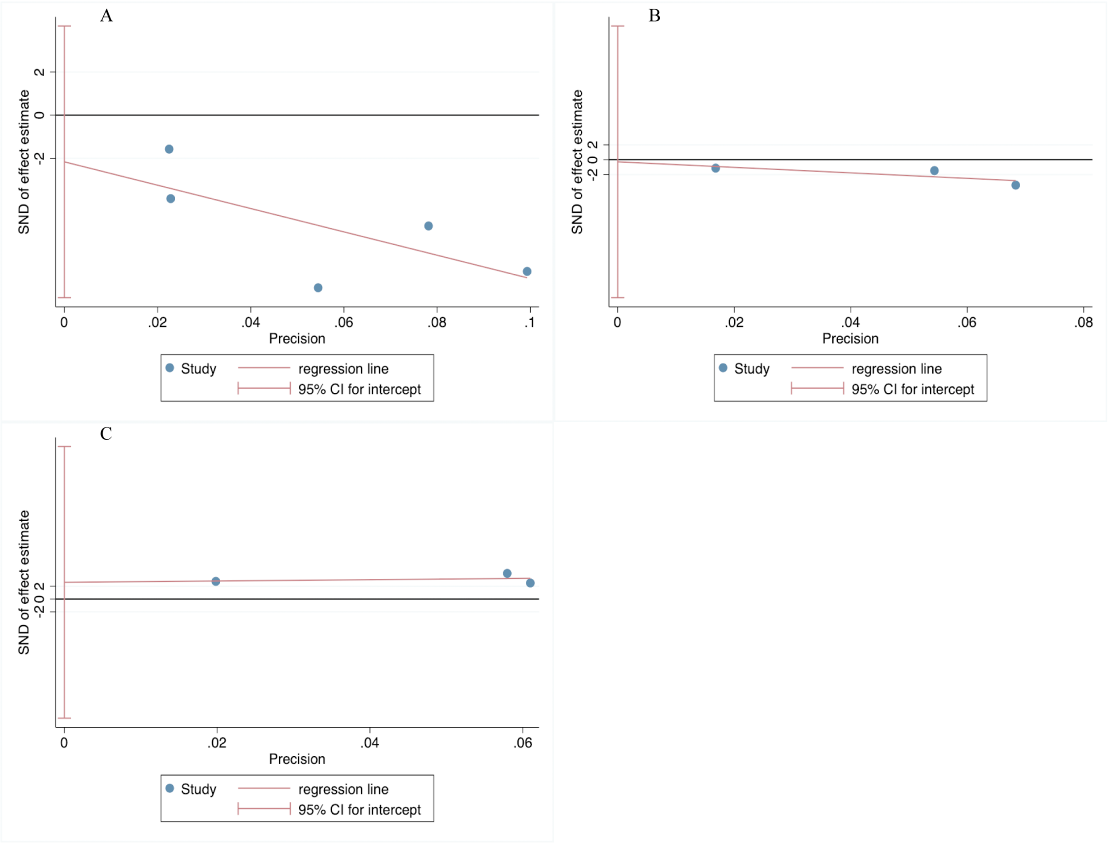

A: DKD versus HV; B: Microalbuminuria DKD versus HV; C:DKD group with eGFR ≥60 ml/min/l.73m^2 versus DKD group with eGFR<60 ml/min/l.73m^2

ADC:

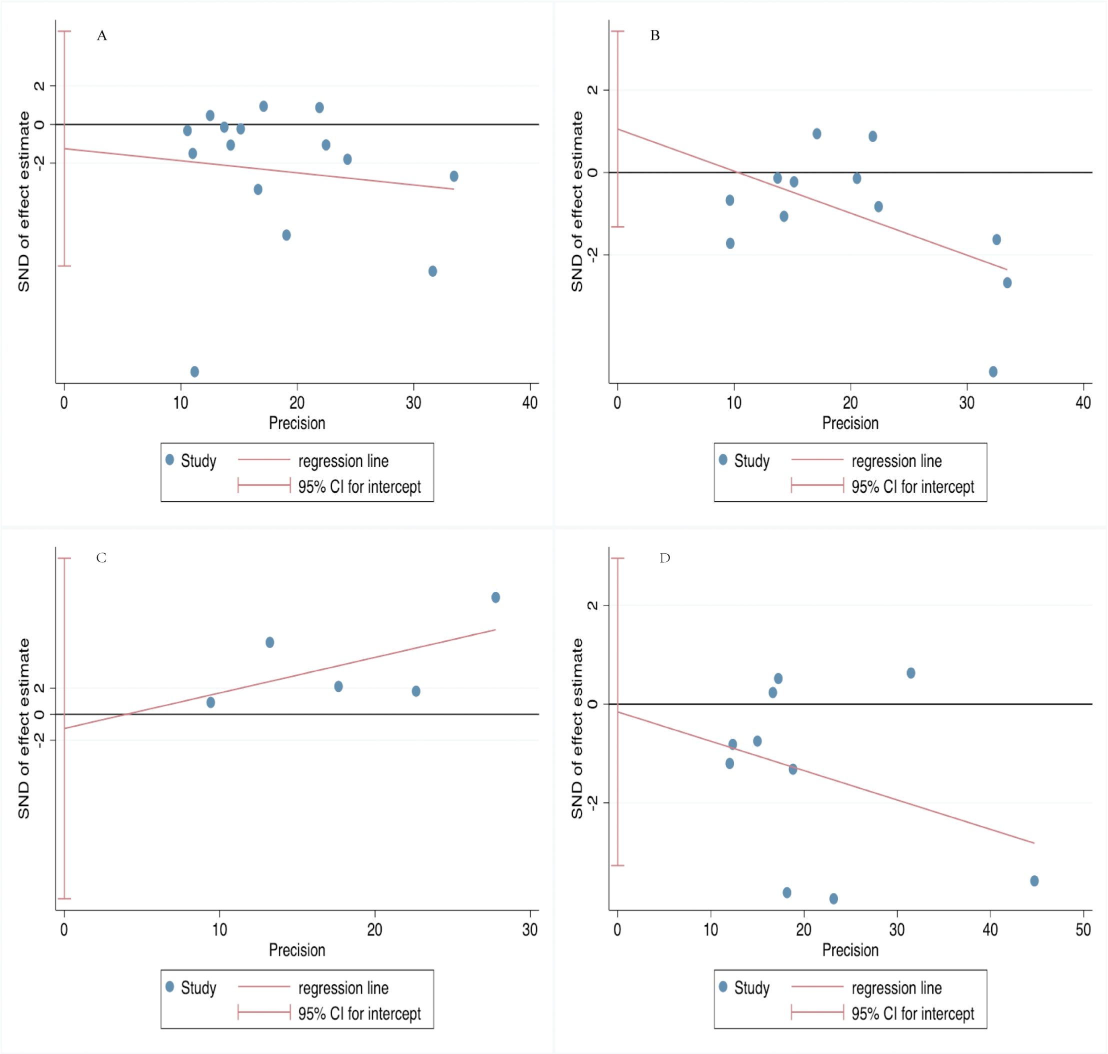

A: DKD versus HV; B:DKD versus simple DM; C: DKD group with eGFR≥60 ml/min/l.73m^2 versus DKD group with eGFR<60 ml/min/l.73m^2; D: DKD versus HV in renal cortex

FA:

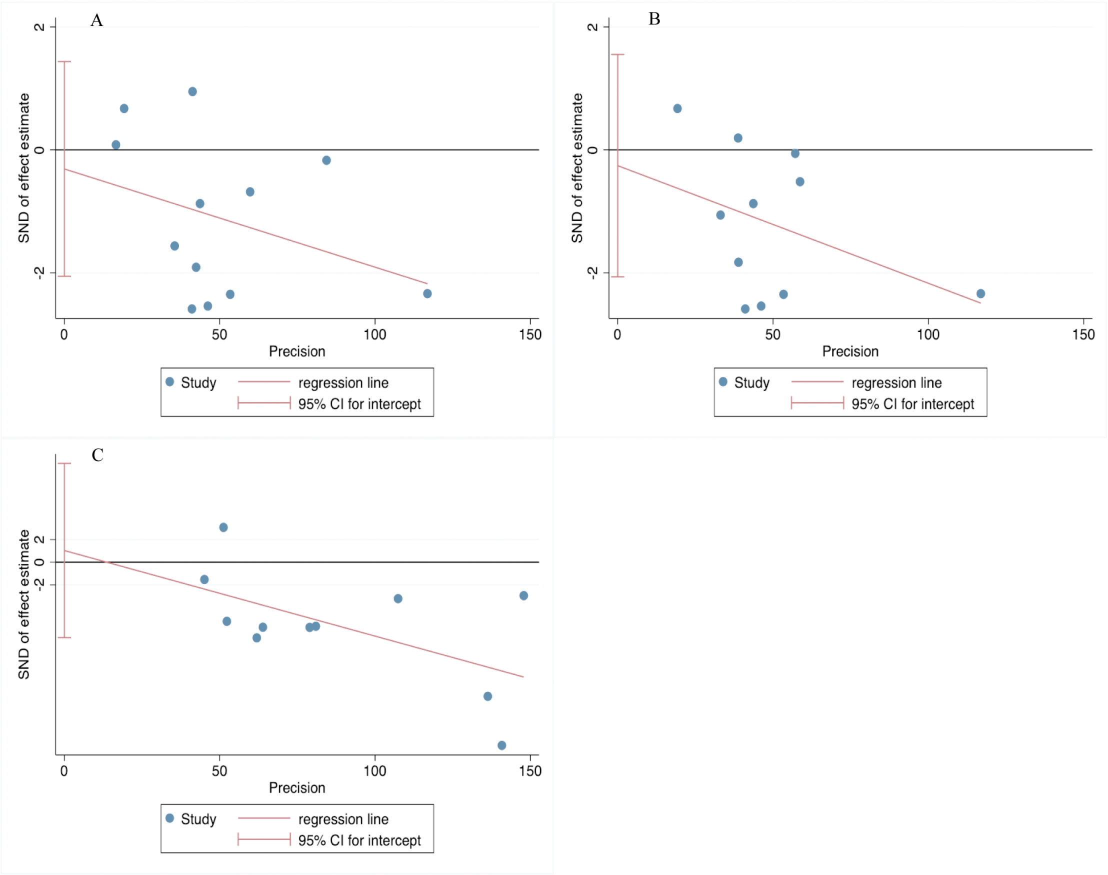

A: DKD versus HV; B: Microalbuminuria DKD versus HV; C: DKD versus HV in renal medulla

f:

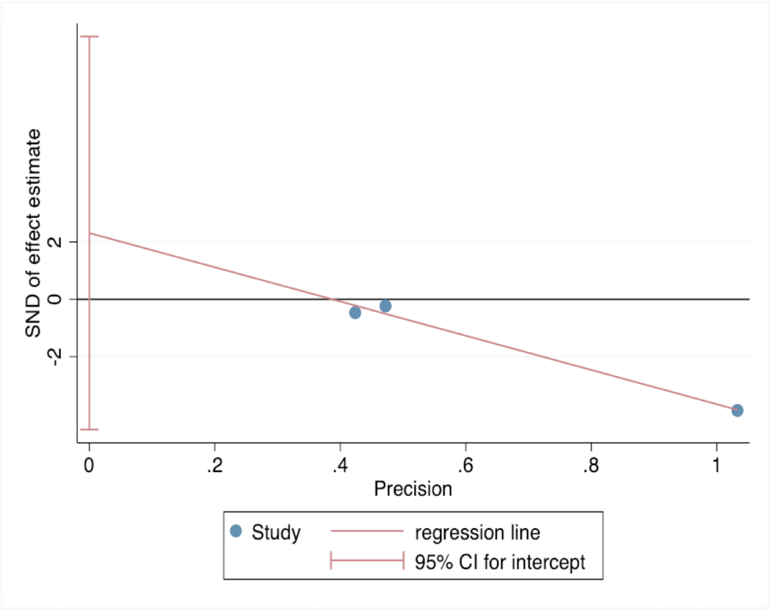

DKD versus simple DM

D:

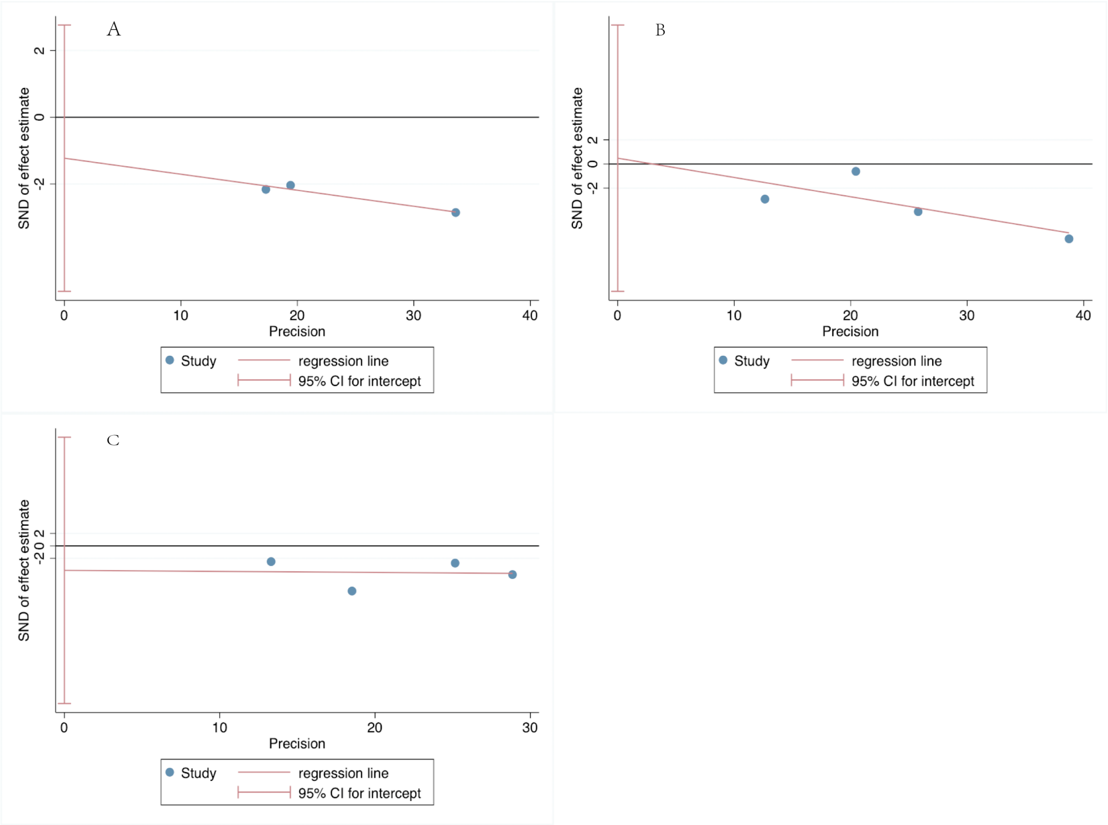

A: DKD versus simple DM; B: DKD group with eGFR≥60 ml/min/l.73m[2] versus DKD group with eGFR<60 ml/min/l.73m[2]; C: DKD versus HV in renal cortex

Figure S5. Forest Plots

RBF:

A

Study or Subgroup	DKD Mean	DKD SD	DKD Total	HV Mean	HV SD	HV Total	Weight	Mean Difference IV, Random, 95% CI
Laursen 2022	360	130	15	430	113	15	11.2%	−70.00 [−157.17, 17.17]
Brown 2020	309.868	116.873	28	479.5	136.471	13	11.4%	−169.63 [−255.52, −83.74]
Makvandi 2022	311	83	36	458	54	20	23.4%	−147.00 [−182.99, −111.01]
Liu 2017	203.957	54.639	25	269.71	33.28	25	26.4%	−65.75 [−90.83, −40.67]
Mora-Gutiérrez 2017	185.842	55.248	44	258.83	37.96	45	27.7%	−72.99 [−92.72, −53.25]
**Total (95% CI)**			**148**			**118**	**100.0%**	**−99.03 [−135.80, −62.27]**

Heterogeneity: $Tau^2 = 1169.63$; $Chi^2 = 18.92$, df = 4 (P = 0.0008); $I^2 = 79\%$
Test for overall effect: Z = 5.28 (P < 0.00001)

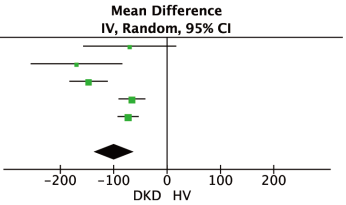

B

Study or Subgroup	DKD Mean	DKD SD	DKD Total	DM Mean	DM SD	DM Total	Weight	Mean Difference IV, Random, 95% CI
Brown 2020	309.868	116.873	28	592.5	12.021	2	49.6%	−282.63 [−329.02, −236.25]
Liu 2017	203.957	54.639	25	258.52	42.3	25	50.4%	−54.56 [−81.65, −27.48]
**Total (95% CI)**			**53**			**27**	**100.0%**	**−167.79 [−391.29, 55.71]**

Heterogeneity: $Tau^2 = 25632.20$; $Chi^2 = 69.25$, df = 1 (P < 0.00001); $I^2 = 99\%$
Test for overall effect: Z = 1.47 (P = 0.14)

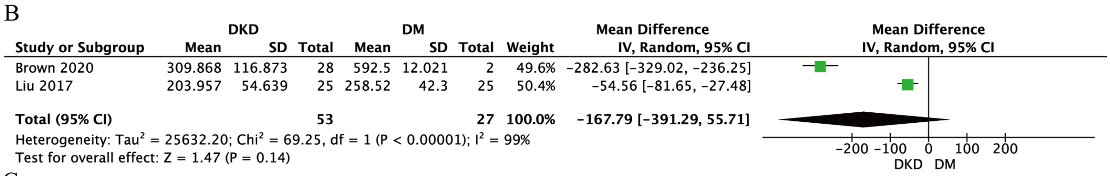

C

Study or Subgroup	DKD Mean	DKD SD	DKD Total	HV Mean	HV SD	HV Total	Weight	Mean Difference IV, Fixed, 95% CI
Brown 2020	412.25	129.739	8	479.5	136.471	13	3.6%	−67.25 [−183.81, 49.31]
Liu 2017	242.86	56.86	11	269.71	33.28	25	37.4%	−26.85 [−62.90, 9.20]
Mora-Gutiérrez 2017	208.89	58.83	19	258.83	37.96	45	59.0%	−49.94 [−78.62, −21.26]
**Total (95% CI)**			**38**			**83**	**100.0%**	**−41.93 [−63.97, −19.89]**

Heterogeneity: $Chi^2 = 1.15$, df = 2 (P = 0.56); $I^2 = 0\%$
Test for overall effect: Z = 3.73 (P = 0.0002)

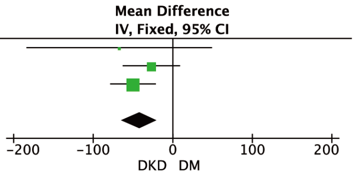

D

Study or Subgroup	eGFR≥60 Mean	eGFR≥60 SD	eGFR≥60 Total	eGFR<60 Mean	eGFR<60 SD	eGFR<60 Total	Weight	Mean Difference IV, Fixed, 95% CI
Brown 2020	412.25	129.739	8	272.4	93.817	20	5.3%	139.85 [40.99, 238.71]
Liu 2017	242.86	56.86	11	173.39	7.16	14	45.0%	69.47 [35.66, 103.28]
Mora-Gutiérrez 2017	208.89	58.83	19	167.597	45.598	24	49.8%	41.29 [9.16, 73.43]
**Total (95% CI)**			**38**			**58**	**100.0%**	**59.15 [36.47, 81.82]**

Heterogeneity: $Chi^2 = 4.10$, df = 2 (P = 0.13); $I^2 = 51\%$
Test for overall effect: Z = 5.11 (P < 0.00001)

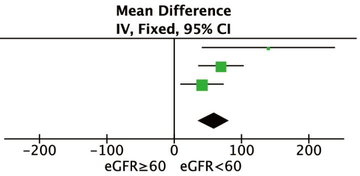

A: DKD versus HV; B: DKD versus simple DM; C: Microalbuminuria DKD versus HV; D: DKD group with eGFR≥ 60 ml/min/1.73m² versus DKD group with eGFR<60 ml/min/1.73m²

R2*:

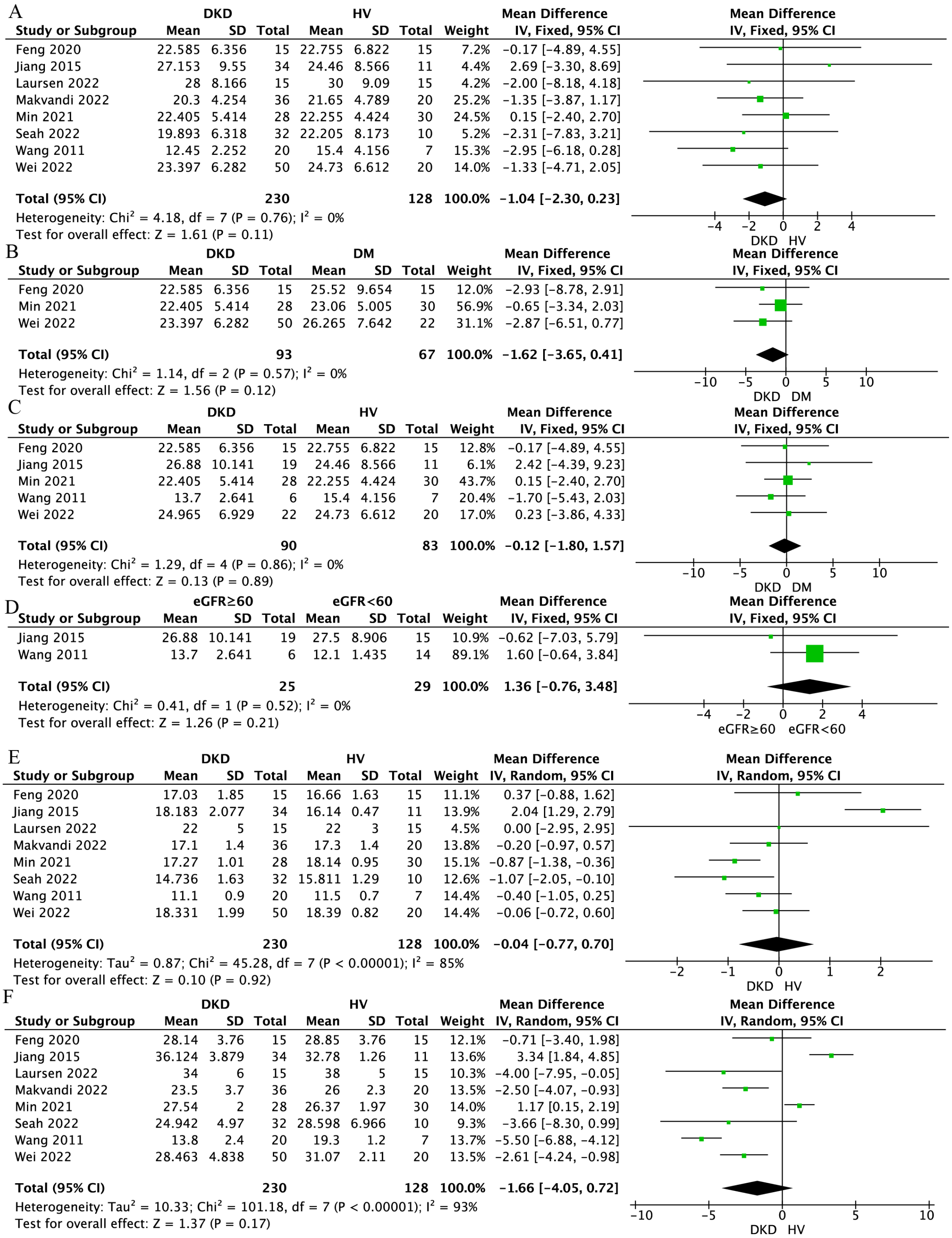

A

Study or Subgroup	DKD Mean	DKD SD	DKD Total	HV Mean	HV SD	HV Total	Weight	Mean Difference IV, Fixed, 95% CI
Feng 2020	22.585	6.356	15	22.755	6.822	15	7.2%	−0.17 [−4.89, 4.55]
Jiang 2015	27.153	9.55	34	24.46	8.566	11	4.4%	2.69 [−3.30, 8.69]
Laursen 2022	28	8.166	15	30	9.09	15	4.2%	−2.00 [−8.18, 4.18]
Makvandi 2022	20.3	4.254	36	21.65	4.789	20	25.2%	−1.35 [−3.87, 1.17]
Min 2021	22.405	5.414	28	22.255	4.424	30	24.5%	0.15 [−2.40, 2.70]
Seah 2022	19.893	6.318	32	22.205	8.173	10	5.2%	−2.31 [−7.83, 3.21]
Wang 2011	12.45	2.252	20	15.4	4.156	7	15.3%	−2.95 [−6.18, 0.28]
Wei 2022	23.397	6.282	50	24.73	6.612	20	14.0%	−1.33 [−4.71, 2.05]
**Total (95% CI)**			**230**			**128**	**100.0%**	**−1.04 [−2.30, 0.23]**

Heterogeneity: $Chi^2 = 4.18$, df = 7 (P = 0.76); $I^2 = 0\%$
Test for overall effect: Z = 1.61 (P = 0.11)

B

Study or Subgroup	DKD Mean	DKD SD	DKD Total	DM Mean	DM SD	DM Total	Weight	Mean Difference IV, Fixed, 95% CI
Feng 2020	22.585	6.356	15	25.52	9.654	15	12.0%	−2.93 [−8.78, 2.91]
Min 2021	22.405	5.414	28	23.06	5.005	30	56.9%	−0.65 [−3.34, 2.03]
Wei 2022	23.397	6.282	50	26.265	7.642	22	31.1%	−2.87 [−6.51, 0.77]
**Total (95% CI)**			**93**			**67**	**100.0%**	**−1.62 [−3.65, 0.41]**

Heterogeneity: $Chi^2 = 1.14$, df = 2 (P = 0.57); $I^2 = 0\%$
Test for overall effect: Z = 1.56 (P = 0.12)

C

Study or Subgroup	DKD Mean	DKD SD	DKD Total	HV Mean	HV SD	HV Total	Weight	Mean Difference IV, Fixed, 95% CI
Feng 2020	22.585	6.356	15	22.755	6.822	15	12.8%	−0.17 [−4.89, 4.55]
Jiang 2015	26.88	10.141	19	24.46	8.566	11	6.1%	2.42 [−4.39, 9.23]
Min 2021	22.405	5.414	28	22.255	4.424	30	43.7%	0.15 [−2.40, 2.70]
Wang 2011	13.7	2.641	6	15.4	4.156	7	20.4%	−1.70 [−5.43, 2.03]
Wei 2022	24.965	6.929	22	24.73	6.612	20	17.0%	0.23 [−3.86, 4.33]
**Total (95% CI)**			**90**			**83**	**100.0%**	**−0.12 [−1.80, 1.57]**

Heterogeneity: $Chi^2 = 1.29$, df = 4 (P = 0.86); $I^2 = 0\%$
Test for overall effect: Z = 0.13 (P = 0.89)

D

Study or Subgroup	eGFR≥60 Mean	eGFR≥60 SD	eGFR≥60 Total	eGFR<60 Mean	eGFR<60 SD	eGFR<60 Total	Weight	Mean Difference IV, Fixed, 95% CI
Jiang 2015	26.88	10.141	19	27.5	8.906	15	10.9%	−0.62 [−7.03, 5.79]
Wang 2011	13.7	2.641	6	12.1	1.435	14	89.1%	1.60 [−0.64, 3.84]
**Total (95% CI)**			**25**			**29**	**100.0%**	**1.36 [−0.76, 3.48]**

Heterogeneity: $Chi^2 = 0.41$, df = 1 (P = 0.52); $I^2 = 0\%$
Test for overall effect: Z = 1.26 (P = 0.21)

E

Study or Subgroup	DKD Mean	DKD SD	DKD Total	HV Mean	HV SD	HV Total	Weight	Mean Difference IV, Random, 95% CI
Feng 2020	17.03	1.85	15	16.66	1.63	15	11.1%	0.37 [−0.88, 1.62]
Jiang 2015	18.183	2.077	34	16.14	0.47	11	13.9%	2.04 [1.29, 2.79]
Laursen 2022	22	5	15	22	3	15	4.5%	0.00 [−2.95, 2.95]
Makvandi 2022	17.1	1.4	36	17.3	1.4	20	13.8%	−0.20 [−0.97, 0.57]
Min 2021	17.27	1.01	28	18.14	0.95	30	15.1%	−0.87 [−1.38, −0.36]
Seah 2022	14.736	1.63	32	15.811	1.29	10	12.6%	−1.07 [−2.05, −0.10]
Wang 2011	11.1	0.9	20	11.5	0.7	7	14.4%	−0.40 [−1.05, 0.25]
Wei 2022	18.331	1.99	50	18.39	0.82	20	14.4%	−0.06 [−0.72, 0.60]
**Total (95% CI)**			**230**			**128**	**100.0%**	**−0.04 [−0.77, 0.70]**

Heterogeneity: $Tau^2 = 0.87$; $Chi^2 = 45.28$, df = 7 (P < 0.00001); $I^2 = 85\%$
Test for overall effect: Z = 0.10 (P = 0.92)

F

Study or Subgroup	DKD Mean	DKD SD	DKD Total	HV Mean	HV SD	HV Total	Weight	Mean Difference IV, Random, 95% CI
Feng 2020	28.14	3.76	15	28.85	3.76	15	12.1%	−0.71 [−3.40, 1.98]
Jiang 2015	36.124	3.879	34	32.78	1.26	11	13.6%	3.34 [1.84, 4.85]
Laursen 2022	34	6	15	38	5	15	10.3%	−4.00 [−7.95, −0.05]
Makvandi 2022	23.5	3.7	36	26	2.3	20	13.5%	−2.50 [−4.07, −0.93]
Min 2021	27.54	2	28	26.37	1.97	30	14.0%	1.17 [0.15, 2.19]
Seah 2022	24.942	4.97	32	28.598	6.966	10	9.3%	−3.66 [−8.30, 0.99]
Wang 2011	13.8	2.4	20	19.3	1.2	7	13.7%	−5.50 [−6.88, −4.12]
Wei 2022	28.463	4.838	50	31.07	2.11	20	13.5%	−2.61 [−4.24, −0.98]
**Total (95% CI)**			**230**			**128**	**100.0%**	**−1.66 [−4.05, 0.72]**

Heterogeneity: $Tau^2 = 10.33$; $Chi^2 = 101.18$, df = 7 (P < 0.00001); $I^2 = 93\%$
Test for overall effect: Z = 1.37 (P = 0.17)

A: DKD versus HV; B: DKD versus simple DM; C: Microalbuminuria DKD versus HV; D: DKD group with eGFR≥60 ml/min/l.73m² versus DKD group with eGFR<60 ml/min/l.73m²; E: DKD versus HV in renal cortex; F: DKD versus HV in renal medulla

ADC:

A

Study or Subgroup	DKD Mean	DKD SD	DKD Total	HV Mean	HV SD	HV Total	Weight	Mean Difference IV, Random, 95% CI
Cakmak 2014	2.09	0.222	78	2.33	0.09	22	7.2%	−0.24 [−0.30, −0.18]
Chen 2014	2.0425	0.1648	16	2.117	0.212	14	6.5%	−0.07 [−0.21, 0.06]
Chen 2018	2.355	0.25	20	2.37	0.183	27	6.6%	−0.02 [−0.14, 0.11]
Feng 2018	1.95	0.092	20	2.03	0.097	20	7.3%	−0.08 [−0.14, −0.02]
Feng 2020	1.96	0.15	15	1.97	0.239	15	6.5%	−0.01 [−0.15, 0.13]
Lu 2011	2.287	0.254	16	2.423	0.145	5	6.0%	−0.14 [−0.31, 0.04]
Makvandi 2022	2.25	0.232	36	2.452	0.206	20	6.7%	−0.20 [−0.32, −0.08]
Mohamed Osman 2021	2.1	0.3	40	2.4	0.1	20	6.9%	−0.30 [−0.40, −0.20]
Mrđanin 2021	2.433	0.265	91	2.48	0.11	10	7.0%	−0.05 [−0.13, 0.04]
Panduranga 2022	2.164	0.446	54	3.307	0.341	27	6.1%	−1.14 [−1.32, −0.97]
Saini 2018	1.797	0.184	60	1.871	0.184	30	7.1%	−0.07 [−0.15, 0.01]
Seah 2022	2.405	0.274	32	2.368	0.201	10	6.3%	0.04 [−0.12, 0.19]
Wang 2018	2.415	0.229	21	2.36	0.177	34	6.8%	0.06 [−0.06, 0.17]
Wei 2022	2.27	0.322	50	2.3	0.371	20	5.9%	−0.03 [−0.22, 0.16]
Ye 2019	2	0.178	36	1.96	0.177	26	7.0%	0.04 [−0.05, 0.13]
**Total (95% CI)**			**585**			**300**	**100.0%**	**−0.14 [−0.24, −0.04]**

Heterogeneity: Tau² = 0.04; Chi² = 195.97, df = 14 (P < 0.00001); I² = 93%
Test for overall effect: Z = 2.79 (P = 0.005)

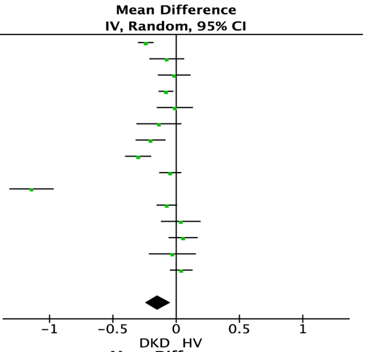

B

Study or Subgroup	DKD Mean	DKD SD	DKD Total	DM Mean	DM SD	DM Total	Weight	Mean Difference IV, Random, 95% CI
Chen 2014	2.0425	0.1648	16	2.098	0.2027	14	15.0%	−0.06 [−0.19, 0.08]
Chen 2018	2.355	0.25	20	2.445	0.234	32	14.8%	−0.09 [−0.23, 0.05]
Feng 2018	1.95	0.092	20	1.985	0.109	20	19.8%	−0.04 [−0.10, 0.03]
Feng 2020	1.96	0.15	15	1.895	0.328	15	11.8%	0.06 [−0.12, 0.25]
Panduranga 2022	2.164	0.446	54	2.724	0.471	19	8.7%	−0.56 [−0.80, −0.32]
Wang 2018	2.415	0.229	21	2.435	0.2001	40	16.2%	−0.02 [−0.14, 0.10]
Wei 2022	2.27	0.322	50	2.225	0.291	22	13.8%	0.04 [−0.11, 0.20]
**Total (95% CI)**			**196**			**162**	**100.0%**	**−0.07 [−0.16, 0.03]**

Heterogeneity: Tau² = 0.01; Chi² = 20.75, df = 6 (P = 0.002); I² = 71%
Test for overall effect: Z = 1.42 (P = 0.16)

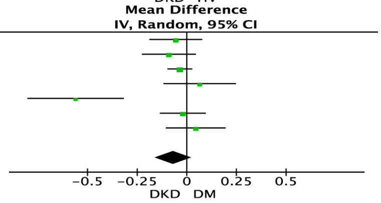

C

Study or Subgroup	DKD Mean	DKD SD	DKD Total	HV Mean	HV SD	HV Total	Weight	Mean Difference IV, Fixed, 95% CI
Cakmak 2014	2.28	0.14	34	2.33	0.09	22	18.5%	−0.05 [−0.11, 0.01]
Chen 2014	2.0425	0.1648	16	2.117	0.212	14	3.6%	−0.07 [−0.21, 0.06]
Chen 2018	2.355	0.25	20	2.37	0.183	27	4.0%	−0.02 [−0.14, 0.11]
Feng 2018	1.95	0.092	20	2.03	0.097	20	19.6%	−0.08 [−0.14, −0.02]
Feng 2020	1.96	0.15	15	1.97	0.239	15	3.3%	−0.01 [−0.15, 0.13]
Lu 2011	2.245	0.255	10	2.423	0.145	5	1.6%	−0.18 [−0.38, 0.02]
Mohamed Osman 2021	2.25	0.086	16	2.4	0.1	20	18.2%	−0.15 [−0.21, −0.09]
Mrđanin 2021	2.473	0.266	61	2.48	0.11	10	7.4%	−0.01 [−0.10, 0.09]
Saini 2018	1.834	0.138	22	1.871	0.184	30	8.8%	−0.04 [−0.12, 0.05]
Wang 2018	2.415	0.229	21	2.36	0.177	34	5.1%	0.06 [−0.06, 0.17]
Wei 2022	2.23	0.293	22	2.3	0.371	20	1.6%	−0.07 [−0.27, 0.13]
Ye 2019	2	0.178	36	1.96	0.177	26	8.4%	0.04 [−0.05, 0.13]
**Total (95% CI)**			**293**			**243**	**100.0%**	**−0.06 [−0.08, −0.03]**

Heterogeneity: Chi² = 21.33, df = 11 (P = 0.03); I² = 48%
Test for overall effect: Z = 4.34 (P < 0.0001)

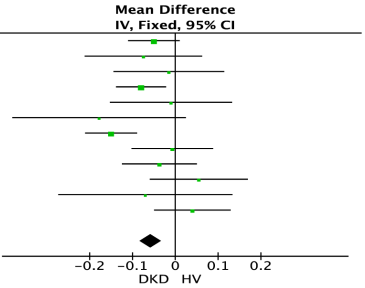

D

Study or Subgroup	eGFR≥60 Mean	eGFR≥60 SD	eGFR≥60 Total	eGFR<60 Mean	eGFR<60 SD	eGFR<60 Total	Weight	Mean Difference IV, Random, 95% CI
Cakmak 2014	2.28	0.14	34	1.957	0.178	44	22.8%	0.32 [0.25, 0.39]
Lu 2011	2.245	0.255	10	2.149	0.169	6	15.4%	0.10 [−0.11, 0.30]
Mohamed Osman 2021	2.25	0.086	16	1.833	0.355	24	18.8%	0.42 [0.27, 0.57]
Mrđanin 2021	2.473	0.266	61	2.352	0.248	30	20.9%	0.12 [0.01, 0.23]
Saini 2018	1.834	0.138	22	1.756	0.203	38	22.1%	0.08 [−0.01, 0.16]
**Total (95% CI)**			**143**			**142**	**100.0%**	**0.21 [0.07, 0.34]**

Heterogeneity: Tau² = 0.02; Chi² = 29.73, df = 4 (P < 0.00001); I² = 87%
Test for overall effect: Z = 3.05 (P = 0.002)

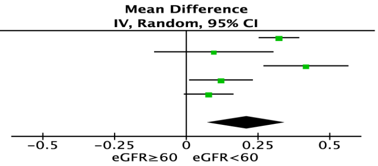

E

Study or Subgroup	DKD Mean	DKD SD	DKD Total	HV Mean	HV SD	HV Total	Weight	Mean Difference IV, Random, 95% CI
Chen 2018	2.39	0.26	20	2.44	0.17	27	8.1%	−0.05 [−0.18, 0.08]
Feng 2018	2.01	0.08	20	2.09	0.06	20	15.5%	−0.08 [−0.12, −0.04]
Feng 2020	2.04	0.1	15	2.11	0.18	15	10.1%	−0.07 [−0.17, 0.03]
Lu 2011	2.441	0.214	16	2.507	0.136	5	6.4%	−0.07 [−0.22, 0.09]
Makvandi 2022	2.31	0.21	36	2.52	0.19	20	9.8%	−0.21 [−0.32, −0.10]
Saini 2018	1.753	0.193	60	1.923	0.193	30	11.8%	−0.17 [−0.25, −0.09]
Seah 2022	2.47	0.217	32	2.456	0.1462	10	9.0%	0.01 [−0.10, 0.13]
Wang 2018	2.44	0.22	21	2.41	0.19	34	9.3%	0.03 [−0.08, 0.14]
Wei 2022	2.45	0.3	50	2.55	0.32	20	6.2%	−0.10 [−0.26, 0.06]
Ye 2019	2.11	0.14	36	2.09	0.11	26	13.9%	0.02 [−0.04, 0.08]
**Total (95% CI)**			**306**			**207**	**100.0%**	**−0.07 [−0.12, −0.02]**

Heterogeneity: Tau² = 0.00; Chi² = 25.11, df = 9 (P = 0.003); I² = 64%
Test for overall effect: Z = 2.62 (P = 0.009)

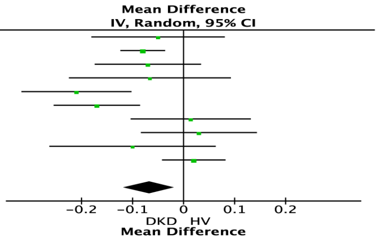

F

Study or Subgroup	DKD Mean	DKD SD	DKD Total	HV Mean	HV SD	HV Total	Weight	Mean Difference IV, Random, 95% CI
Chen 2018	2.32	0.24	20	2.3	0.17	27	8.9%	0.02 [−0.10, 0.14]
Feng 2018	1.89	0.06	20	1.97	0.09	20	13.9%	−0.08 [−0.13, −0.03]
Feng 2020	1.88	0.15	15	1.83	0.21	15	8.5%	0.05 [−0.08, 0.18]
Lu 2011	2.132	0.191	16	2.338	0.104	5	8.5%	−0.21 [−0.34, −0.08]
Makvandi 2022	2.19	0.24	36	2.33	0.18	20	9.7%	−0.14 [−0.25, −0.03]
Saini 2018	1.841	0.162	60	1.818	0.162	30	12.4%	0.02 [−0.05, 0.09]
Seah 2022	2.339	0.31	32	2.279	0.215	10	6.4%	0.06 [−0.11, 0.23]
Wang 2018	2.39	0.24	21	2.31	0.15	34	9.5%	0.08 [−0.03, 0.19]
Wei 2022	2.09	0.23	50	2.05	0.22	20	9.4%	0.04 [−0.08, 0.16]
Ye 2019	1.89	0.14	36	1.83	0.13	26	12.6%	0.06 [−0.01, 0.13]
**Total (95% CI)**			**306**			**207**	**100.0%**	**−0.01 [−0.07, 0.05]**

Heterogeneity: Tau² = 0.01; Chi² = 31.50, df = 9 (P = 0.0002); I² = 71%
Test for overall effect: Z = 0.36 (P = 0.72)

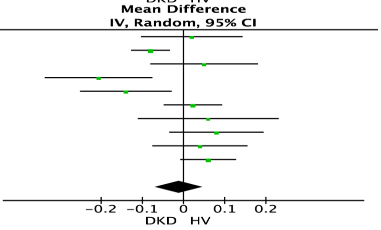

A: DKD versus HV; B: DKD versus simple DM; C: Microalbuminuria DKD versus HV; D: DKD group with eGFR≥60 ml/min/l.73m2 versus DKD group with eGFR<60 ml/min/l.73m2; E: DKD versus HV in renal cortex; F: DKD versus HV in renal medulla

FA:

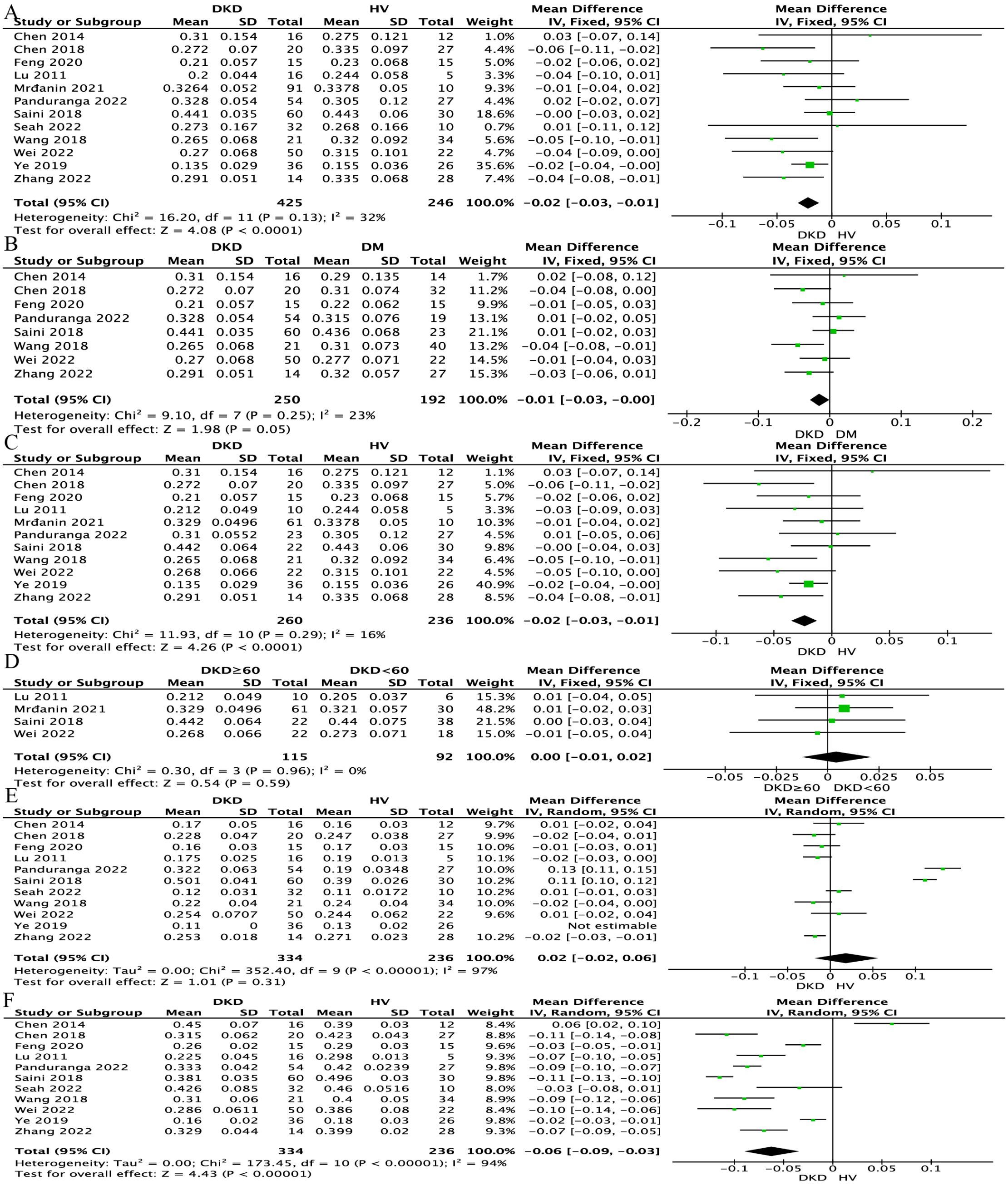

A: DKD versus HV; B: DKD versus simple DM; C: Microalbuminuria DKD versus HV; D: DKD group with eGFR≥60 ml/min/l.73m² versus DKD group with eGFR<60 ml/min/l.73m²; E: DKD versus HV in renal cortex; F: DKD versus HV in renal medulla

f:

A

Study or Subgroup	DKD Mean	DKD SD	DKD Total	HV Mean	HV SD	HV Total	Weight	Mean Difference IV, Random, 95% CI
Chen 2018	17.4	3.158	20	21.75	2.956	27	23.0%	−4.35 [−6.13, −2.57]
Deng 2018	23.705	5.478	19	29.195	6.891	12	18.2%	−5.49 [−10.10, −0.88]
Feng 2018	26.635	6.637	20	21.74	5.227	20	19.9%	4.90 [1.19, 8.60]
Makvandi 2022	14.85	5.671	36	12.9	5.373	20	21.2%	1.95 [−1.05, 4.95]
Zhang 2022	29.1	7.697	14	32.5	7.014	28	17.8%	−3.40 [−8.20, 1.40]
**Total (95% CI)**			**109**			**107**	**100.0%**	**−1.21 [−5.19, 2.76]**

Heterogeneity: $Tau^2 = 17.06$; $Chi^2 = 29.24$, df = 4 (P < 0.00001); $I^2 = 86\%$

Test for overall effect: Z = 0.60 (P = 0.55)

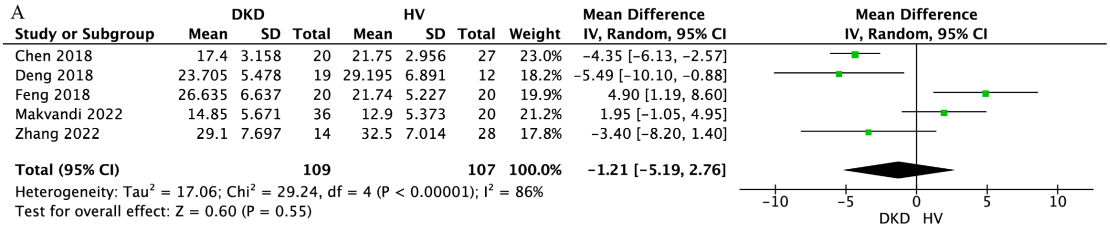

Study or Subgroup	Mean	SD	Total	Mean	SD	Total	Weight	IV, Fixed, 95% CI
Chen 2018	17.4	3.158	20	21.15	3.747	32	72.6%	−3.75 [−5.65, −1.85]
Feng 2018	26.635	6.637	20	27.13	6.763	20	15.2%	−0.49 [−4.65, 3.66]
Zhang 2022	29.1	7.697	14	30.2	6.013	27	12.2%	−1.10 [−5.73, 3.53]
**Total (95% CI)**			**54**			**79**	**100.0%**	**−2.93 [−4.55, −1.32]**

Heterogeneity: $Chi^2 = 2.64$, df = 2 (P = 0.27); $I^2 = 24\%$

Test for overall effect: Z = 3.55 (P = 0.0004)

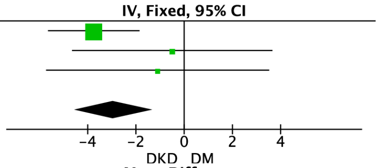

C

Study or Subgroup	DKD Mean	DKD SD	DKD Total	HV Mean	HV SD	HV Total	Weight	Mean Difference IV, Random, 95% CI
Chen 2018	17.4	3.158	20	21.75	2.956	27	28.8%	−4.35 [−6.13, −2.57]
Deng 2018	23.705	5.478	19	29.195	6.891	12	23.2%	−5.49 [−10.10, −0.88]
Feng 2018	26.635	6.637	20	21.74	5.227	20	25.2%	4.90 [1.19, 8.60]
Zhang 2022	29.1	7.697	14	32.5	7.014	28	22.7%	−3.40 [−8.20, 1.40]
**Total (95% CI)**			**73**			**87**	**100.0%**	**−2.06 [−6.68, 2.56]**

Heterogeneity: $Tau^2 = 18.45$; $Chi^2 = 20.83$, df = 3 (P = 0.0001); $I^2 = 86\%$

Test for overall effect: Z = 0.88 (P = 0.38)

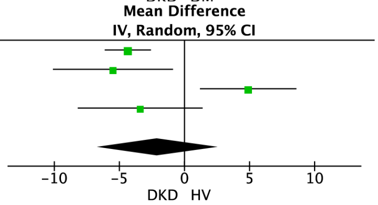

D

Study or Subgroup	DKD Mean	DKD SD	DKD Total	HV Mean	HV SD	HV Total	Weight	Mean Difference IV, Random, 95% CI
Chen 2018	19	3.2	20	23.9	2.5	27	26.3%	−4.90 [−6.59, −3.21]
Feng 2018	32.1	3.63	19	23.95	4.55	12	25.3%	8.15 [5.10, 11.20]
Makvandi 2022	15.2	5.7	20	13.5	5.7	20	24.8%	1.70 [−1.83, 5.23]
Zhang 2022	31.8	8.3	36	34	8.4	20	23.6%	−2.20 [−6.77, 2.37]
**Total (95% CI)**			**95**			**79**	**100.0%**	**0.67 [−5.84, 7.19]**

Heterogeneity: $Tau^2 = 41.33$; $Chi^2 = 57.26$, df = 3 (P < 0.00001); $I^2 = 95\%$

Test for overall effect: Z = 0.20 (P = 0.84)

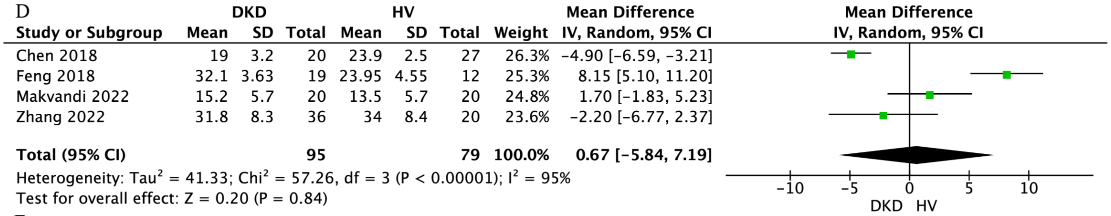

E

Study or Subgroup	DKD Mean	DKD SD	DKD Total	HV Mean	HV SD	HV Total	Weight	Mean Difference IV, Random, 95% CI
Chen 2018	15.8	2.2	20	19.6	1.4	27	29.4%	−3.80 [−4.90, −2.70]
Feng 2018	21.17	3.79	19	19.53	5.01	12	23.2%	1.64 [−1.67, 4.95]
Makvandi 2022	14.5	5.7	20	12.3	5.1	20	23.1%	2.20 [−1.15, 5.55]
Zhang 2022	26.4	6.2	36	31	5	20	24.3%	−4.60 [−7.58, −1.62]
**Total (95% CI)**			**95**			**79**	**100.0%**	**−1.35 [−4.62, 1.92]**

Heterogeneity: $Tau^2 = 9.13$; $Chi^2 = 19.94$, df = 3 (P = 0.0002); $I^2 = 85\%$

Test for overall effect: Z = 0.81 (P = 0.42)

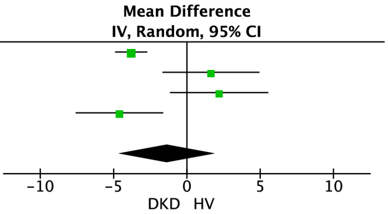

A: DKD versus HV; B: DKD versus simple DM; C: Microalbuminuria DKD versus HV; D: DKD versus HV in renal cortex; E: DKD versus HV in renal medulla

D:

A

Study or Subgroup	DKD Mean	DKD SD	DKD Total	HV Mean	HV SD	HV Total	Weight	Mean Difference IV, Random, 95% CI
Chen 2018	1.78	0.197	20	1.85	0.147	27	20.4%	−0.07 [−0.17, 0.03]
Deng 2018	1.845	0.194	19	1.72	0.155	12	19.0%	0.13 [0.00, 0.25]
Feng 2018	1.58	0.099	20	1.74	0.094	20	22.8%	−0.16 [−0.22, −0.10]
Makvandi 2022	1.88	0.232	36	2.09	0.257	20	18.2%	−0.21 [−0.35, −0.07]
Zhang 2022	1.378	0.187	14	1.651	0.175	28	19.5%	−0.27 [−0.39, −0.16]
**Total (95% CI)**			**109**			**107**	**100.0%**	**−0.12 [−0.23, −0.00]**

Heterogeneity: $Tau^2 = 0.01$; $Chi^2 = 25.63$, df = 4 ($P < 0.0001$); $I^2 = 84\%$
Test for overall effect: Z = 2.00 (P = 0.05)

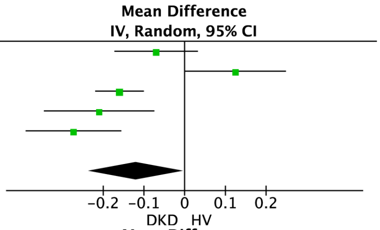

B

Study or Subgroup	DKD Mean	DKD SD	DKD Total	DM Mean	DM SD	DM Total	Weight	Mean Difference IV, Fixed, 95% CI
Chen 2018	1.78	0.197	20	1.885	0.151	32	20.9%	−0.10 [−0.21, −0.00]
Feng 2018	1.58	0.099	20	1.665	0.089	20	62.5%	−0.08 [−0.14, −0.03]
Zhang 2022	1.378	0.187	14	1.503	0.151	27	16.6%	−0.13 [−0.24, −0.01]
**Total (95% CI)**			**54**			**79**	**100.0%**	**−0.10 [−0.14, −0.05]**

Heterogeneity: $Chi^2 = 0.42$, df = 2 ($P = 0.81$); $I^2 = 0\%$
Test for overall effect: Z = 4.07 (P < 0.0001)

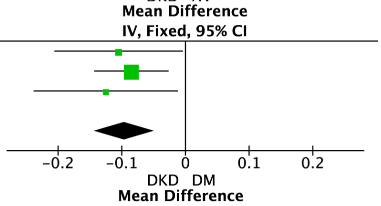

C

Study or Subgroup	DKD Mean	DKD SD	DKD Total	HV Mean	HV SD	HV Total	Weight	Mean Difference IV, Random, 95% CI
Chen 2018	1.78	0.197	20	1.85	0.147	27	25.0%	−0.07 [−0.17, 0.03]
Deng 2018	1.845	0.194	19	1.72	0.155	12	23.5%	0.13 [0.00, 0.25]
Feng 2018	1.58	0.099	20	1.74	0.094	20	27.5%	−0.16 [−0.22, −0.10]
Zhang 2022	1.378	0.187	14	1.651	0.175	28	24.0%	−0.27 [−0.39, −0.16]
**Total (95% CI)**			**73**			**87**	**100.0%**	**−0.10 [−0.24, 0.04]**

Heterogeneity: $Tau^2 = 0.02$; $Chi^2 = 24.20$, df = 3 ($P < 0.0001$); $I^2 = 88\%$
Test for overall effect: Z = 1.39 (P = 0.17)

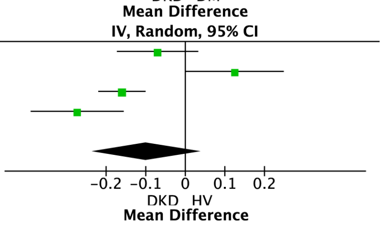

D

Study or Subgroup	DKD Mean	DKD SD	DKD Total	HV Mean	HV SD	HV Total	Weight	Mean Difference IV, Random, 95% CI
Chen 2018	1.89	0.17	20	1.92	0.16	27	23.0%	−0.03 [−0.13, 0.07]
Feng 2018	1.63	0.07	19	1.79	0.07	12	36.0%	−0.16 [−0.21, −0.11]
Makvandi 2022	1.91	0.26	20	2.14	0.24	20	12.7%	−0.23 [−0.39, −0.07]
Zhang 2022	1.488	0.146	36	1.641	0.135	20	28.3%	−0.15 [−0.23, −0.08]
**Total (95% CI)**			**95**			**79**	**100.0%**	**−0.14 [−0.20, −0.07]**

Heterogeneity: $Tau^2 = 0.00$; $Chi^2 = 7.04$, df = 3 ($P = 0.07$); $I^2 = 57\%$
Test for overall effect: Z = 4.13 (P < 0.0001)

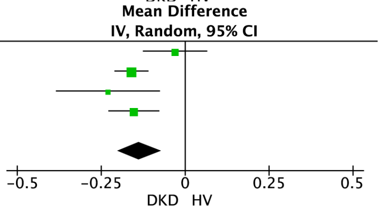

E

Study or Subgroup	DKD Mean	DKD SD	DKD Total	HV Mean	HV SD	HV Total	Weight	Mean Difference IV, Random, 95% CI
Chen 2018	1.67	0.16	20	1.78	0.09	27	27.0%	−0.11 [−0.19, −0.03]
Feng 2018	1.53	0.1	19	1.69	0.09	12	27.8%	−0.16 [−0.23, −0.09]
Makvandi 2022	1.85	0.2	20	2.04	0.27	20	20.6%	−0.19 [−0.34, −0.04]
Zhang 2022	1.268	0.16	36	1.66	0.21	20	24.5%	−0.39 [−0.50, −0.29]
**Total (95% CI)**			**95**			**79**	**100.0%**	**−0.21 [−0.33, −0.09]**

Heterogeneity: $Tau^2 = 0.01$; $Chi^2 = 18.77$, df = 3 ($P = 0.0003$); $I^2 = 84\%$
Test for overall effect: Z = 3.51 (P = 0.0004)

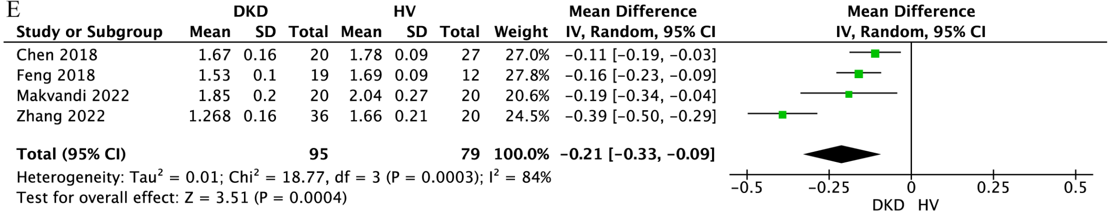

A: DKD versus HV; B: DKD versus simple DM; C: Microalbuminuria DKD versus HV; D: DKD versus HV in renal cortex; E: DKD versus HV in renal medulla

D*:

A

Study or Subgroup	DKD Mean	DKD SD	DKD Total	HV Mean	HV SD	HV Total	Weight	Mean Difference IV, Fixed, 95% CI
Chen 2018	25.5	15.898	20	22.5	9.346	27	6.1%	3.00 [−4.81, 10.81]
Deng 2018	14.525	8.988	19	12.705	8.161	12	9.9%	1.82 [−4.32, 7.96]
Feng 2018	17.505	5.112	20	16.56	4.944	20	38.3%	0.95 [−2.17, 4.06]
Makvandi 2022	144	594.674	36	127	332.306	20	0.0%	17.00 [−225.79, 259.79]
Zhang 2022	14.209	4.42	14	18.363	4.49	28	45.8%	−4.15 [−7.00, −1.30]
**Total (95% CI)**			**109**			**107**	**100.0%**	**−1.18 [−3.10, 0.75]**

Heterogeneity: Chi² = 8.01, df = 4 (P = 0.09); I² = 50%
Test for overall effect: Z = 1.19 (P = 0.23)

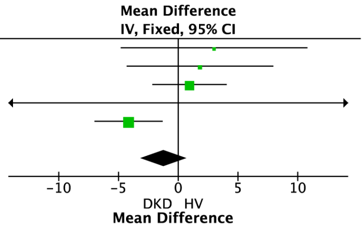

B

Study or Subgroup	DKD Mean	DKD SD	DKD Total	DM Mean	DM SD	DM Total	Weight	Mean Difference IV, Random, 95% CI
Chen 2018	25.5	15.898	20	32.5	22.121	32	12.8%	−7.00 [−17.36, 3.36]
Feng 2018	17.505	5.112	20	17.46	5.33	20	44.0%	0.04 [−3.19, 3.28]
Zhang 2022	14.209	4.42	14	19.105	6.377	27	43.3%	−4.90 [−8.23, −1.56]
**Total (95% CI)**			**54**			**79**	**100.0%**	**−2.99 [−7.16, 1.18]**

Heterogeneity: Tau² = 7.56; Chi² = 5.07, df = 2 (P = 0.08); I² = 61%
Test for overall effect: Z = 1.41 (P = 0.16)

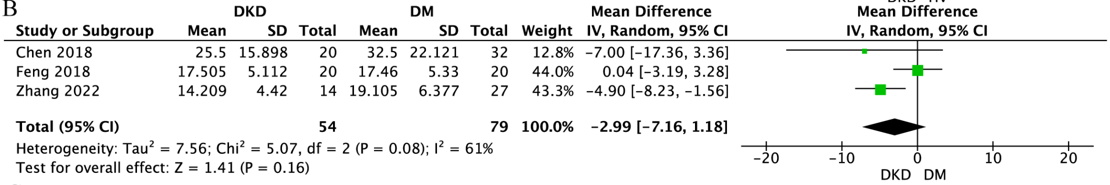

C

Study or Subgroup	DKD Mean	DKD SD	DKD Total	HV Mean	HV SD	HV Total	Weight	Mean Difference IV, Random, 95% CI
Chen 2018	25.5	15.898	20	22.5	9.346	27	14.1%	3.00 [−4.81, 10.81]
Deng 2018	14.525	8.988	19	12.705	8.161	12	19.1%	1.82 [−4.32, 7.96]
Feng 2018	17.505	5.112	20	16.56	4.944	20	32.7%	0.95 [−2.17, 4.06]
Zhang 2022	14.209	4.42	14	18.363	4.49	28	34.1%	−4.15 [−7.00, −1.30]
**Total (95% CI)**			**73**			**87**	**100.0%**	**−0.34 [−3.91, 3.24]**

Heterogeneity: Tau² = 7.65; Chi² = 7.99, df = 3 (P = 0.05); I² = 62%
Test for overall effect: Z = 0.18 (P = 0.85)

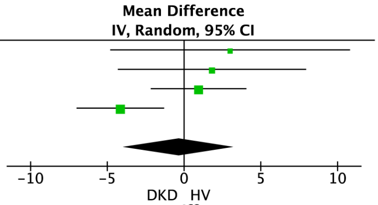

D

Study or Subgroup	DKD Mean	DKD SD	DKD Total	HV Mean	HV SD	HV Total	Weight	Mean Difference IV, Random, 95% CI
Chen 2018	22	13	20	18	6	27	20.0%	4.00 [−2.13, 10.13]
Feng 2018	13.84	4.44	19	13.59	2.55	12	40.5%	0.25 [−2.21, 2.71]
Makvandi 2022	98	351	20	56	73.3	20	0.1%	42.00 [−115.15, 199.15]
Zhang 2022	14.906	5.087	36	18.56	4.648	20	39.4%	−3.65 [−6.28, −1.03]
**Total (95% CI)**			**95**			**79**	**100.0%**	**−0.52 [−4.05, 3.02]**

Heterogeneity: Tau² = 6.44; Chi² = 7.73, df = 3 (P = 0.05); I² = 61%
Test for overall effect: Z = 0.29 (P = 0.77)

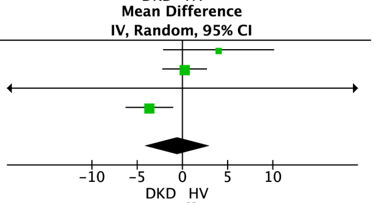

E

Study or Subgroup	DKD Mean	DKD SD	DKD Total	HV Mean	HV SD	HV Total	Weight	Mean Difference IV, Random, 95% CI
Chen 2018	29	18	20	27	10	27	18.9%	2.00 [−6.74, 10.74]
Feng 2018	21.17	3.79	19	19.53	5.01	12	38.6%	1.64 [−1.67, 4.95]
Makvandi 2022	190	768	20	198	459	20	0.0%	−8.00 [−400.12, 384.12]
Zhang 2022	13.512	3.696	36	18.166	4.402	20	42.5%	−4.65 [−6.93, −2.38]
**Total (95% CI)**			**95**			**79**	**100.0%**	**−0.97 [−5.90, 3.96]**

Heterogeneity: Tau² = 13.54; Chi² = 10.47, df = 3 (P = 0.01); I² = 71%
Test for overall effect: Z = 0.38 (P = 0.70)

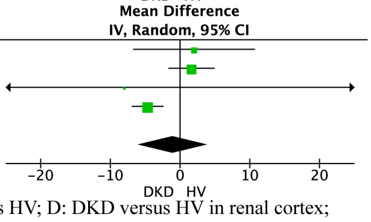

A: DKD versus HV; B: DKD versus simple DM; C: Microalbuminuria DKD versus HV; D: DKD versus HV in renal cortex; E: DKD versus HV in renal medulla
